# Validating the dual evolutionary foundations of political values in a US sample

**DOI:** 10.3389/fpsyg.2023.1189771

**Published:** 2023-06-23

**Authors:** Guy A. Lavender Forsyth, Ananish Chaudhuri, Quentin Douglas Atkinson

**Affiliations:** ^1^School of Psychology, University of Auckland, Auckland, New Zealand; ^2^Department of Economics, University of Auckland, Auckland, New Zealand; ^3^Centre for the Study of Social Cohesion, School of Anthropology and Museum Ethnography, University of Oxford, Oxford, United Kingdom

**Keywords:** politics, values, ideology, evolution, cooperation, authoritarianism, dominance

## Abstract

Psychological research repeatedly identifies two dimensions of political values. Recent work argues that these dimensions reflect the dual evolutionary foundations of human social and political life: a trade-off between cooperation and competition that generates differences in values about social inequality, and a trade-off in managing group coordination that generates differences in values about social control. Existing scales used to measure political values, however, were created prior to this framework. Here, we introduce the Dual Foundations Scale, designed to capture values about the two trade-offs. We validate the scale across two studies, showing it accurately and reliably measures both dimensions. Our results support key predictions of the dual foundations framework and pave the way for future work on the foundations of political ideology.

## 1. Introduction

Values—preferences people have about collective goals and general principles of conduct—are central constructs for understanding human politics. Rather than merely attending to individuals’ own personal goals and aspirations, to study values is to study individuals’ preferences about the way people *ought* to behave and the way things *ought* to be (Schwartz, 2012; Bergh and Sidanius, 2021). Values thus bridge between individuals and social groups, and between individual psychology and politics. Intriguingly, of the great diversity of different values that individuals might hold, much work on the structure of human values identifies two overriding dimensions (Claessens et al., 2020). Converging findings from various studies indicate the two-dimensional structure of values is widespread, including work in politics (Duckitt and Sibley, 2010) as well as studies of the basic structure of personal values (Schwartz, 1992; Schwartz et al., 2012). Yet, despite mounting evidence for two recurring dimensions, until recently there has been no single framework to explain this pattern from first principles. Our recently proposed dual foundations framework offers a solution by grounding the two dimensions in evolutionary trade-offs that are inherent to human social life. Here, we create a scale—the Dual Foundations Scale—that operationalizes the dual foundations framework and validate it as a measure of the two fundamental dimensions of political values.

Two value dimensions are known by different names across a diverse array of work (see Table 1). Schwartz’s research characterizes them, respectively, as tensions between “Self-Enhancement” and “Self-Transcendence” and between “Conservation” and “Openness to Change” (Schwartz, 1992). But they have deeper intellectual histories, as documented by Tomkins (1964) work on “humanism” and “normativism” and Rokeach (1973) on “equality” and “freedom.” They exist too at different levels of social organization (Lakoff, 2016). In Feinberg et al. (2020) study of family values, the two dimensions are named “Nurturant Parent” and “Strict Father.” They also appear in adjacent disciplines, including D’Andrade (2008) anthropological study of “altruism versus self-interest” and “individualism versus collectivism.” And in the discipline of political psychology, where values are studied under the umbrella of ideology, there is growing appreciation that what many considered to be a single ideological dimension is better characterized as two (Duckitt and Sibley, 2010).

**Table 1:** Various definitions for the two dimensions of ideology.

Inequality-type dimension	Social control-type dimension	References
Nurturant parent	Strict Father	Feinberg et al. (2020)
Acceptance of inequality	Rejecting system change	Kandler et al. (2012)
Individualizing (care-harm, fairness-reciprocity)	Binding (authority-respect, in-group-loyalty, purity-sanctity)	Graham et al. (2009)
Altruism vs. self-interest	Individualism vs. collectivism	D’Andrade (2008)
Egalitarianism	Conservatism	Stangor and Leary (2006)
Competition vs. compassion	Moral regulation vs. individual freedom	Ashton et al. (2005)
Tolerance of inequality	Opposition to change	Jost et al. (2003)
Social dominance orientation	Right-wing authoritarianism	Duckitt (2001)
Unmitigated self-interest (“beta-isms”)	Tradition-oriented religiousness (“alpha-isms”)	Saucier (2000)
Vertical vs. horizontal values	Collectivism vs. individualism	Triandis and Gelfand (1998)
International harmony	National strength and order	Braithwaite (1994)
Capitalist vs. socialist	Religious vs. secular	Boski (1993)
Hierarchy vs. egalitarianism	Group loyalty vs. individualism	Trompenaars (1993)
Self-enhancement vs. transcendence	Conservation vs. openness	Schwartz (1992)
Humanitarianism-egalitarianism	Protestant ethic	Katz and Hass (1988)
Liberalism (i.e., humanism-egalitarianism)	Conservatism	Kerlinger (1984)
Idealism (altruism-social concern)	Relativism (i.e., group orientation)	Forsyth (1980)
Power distance	Collectivism vs. individualism	Hofstede (1980)
Equality	Freedom	Rokeach (1973)
Humanism	Normativism (conservatism)	Tomkins (1964)
Tough vs. tender	Conservatism vs. liberalism	Eysenck (1954)
Humanitarianism	Religiosity	Ferguson (1939)

Table data adapted and extended from Duckitt and Sibley (2009) and Claessens et al. (2020).

Though many are still familiar with political ideology arranged in a single dimension from left to right or liberal to conservative, increasing evidence favors the existence of two basic dimensions (Duckitt and Sibley, 2010). Duckitt and Sibley (2009), for example, argue the best representation of politics’ two dimensions is a pair of scales named Social Dominance Orientation (SDO) and Right Wing Authoritarianism (RWA). Whereas SDO measures preferences for unequal relationships between social groups (Ho et al., 2015), RWA measures preferences for three forms of social control: support for traditional norms, for strong leadership, and for punishment of deviance (Duckitt et al., 2010).

Several lines of evidence support the two-dimensional account of political ideology. In factor analyses, more complex models with a greater number of dimensions often reduce to the two dimensions (Federico et al., 2013; Sinn and Hayes, 2017). Each dimension is independently and uniquely associated with other aspects of personality, belief, attitudes, and behavior (Duckitt and Sibley, 2009; Schwartz et al., 2012; Feinberg et al., 2020). Research with SDO and RWA in particular shows them to be both relatively stable across time and to causally precede changes in other behaviors (Sibley and Duckitt, 2013; Osborne et al., 2021; Satherley et al., 2021). Furthermore, cross-national research supports the conceptual independence of the two dimensions, which sometimes correlate positively, sometimes correlate negatively, and which are sometimes uncorrelated (Mirisola et al., 2007; de Regt et al., 2011; Malka et al., 2019). The two dimensions thus constitute independent and enduring psychological constructs with particular importance for political attitudes and behavior. The question is: what explains this recurrent structure?

The dual foundations framework has recently proposed that the various existing two-dimensional measures of values and ideology reflect the independent rediscovering of two fundamental challenges or trade-offs that are inherent to the evolution of human group-living (Claessens et al., 2020): one related to fairness in managing and distributing public goods and the other related to the extent of social control in collective activities (Tomasello et al., 2012; Tomasello and Vaish, 2013; Jensen et al., 2014; Burkart et al., 2018).

The first dimension arises because human social life is highly interdependent (Aktipis et al., 2018) and this requires decisions to be made about how to manage and distribute resources. In turn, such resource allocation decisions generate a conflict between cooperating for the common good (Dugatkin, 1997, p. 11) and competition or free-riding to enhance one’s own payoff at the expense of others (Johnson and Johnson, 2015). Social equality thus aligns more closely with cooperation than competition (Townsend, 2018; Hooper et al., 2021), with competition associated with efforts to improve one’s absolute returns and to enhance one’s own relative standing against others (Hickey and Davidsen, 2019; Mandalaywala, 2019; Wilson and Codding, 2020). Tensions will thus arise between those who favor cooperation and more even resource distributions and those who favor more competitive and uneven resource distributions (Singh et al., 2017; Pandit et al., 2020; Powers et al., 2021), leading to the between-individual differences in values that comprise the inequality dimension.

The second dimension of ideology arises because humans’ reliance on groups of genetic non-relatives selected for a concern for group viability and sophisticated cultural tools for managing and coordinating group living (Bissonnette et al., 2015; Wiessner, 2019). These tools for ensuring coordination and collective activities often emphasize social control, which is traded off against individual autonomy, such as mandating deference of established social norms and leadership (Hawkins et al., 2019; Pietraszewski, 2020) and sanctioning those who deviate (Wiessner, 2020; Eriksson et al., 2021). Nevertheless, diminished autonomy can restrict the ability to innovate and adopt novel solutions to social problems, creating other incentives to loosen social control (Kendal et al., 2005; Whitehead and Richerson, 2009). The dual foundations framework thus expects the trade-off between this second set of strategies will shape individuals’ values about social control and autonomy (Claessens et al., 2020).

If the dual foundations’ explanation for the two dimensions is accurate, there may be a mismatch between what existing scales were created to measure and what they are theorized to represent. By not updating measurement instruments along with theoretical developments, gaps are created between theory-driven hypotheses and the way these hypotheses are tested. Thus, while many scales seem to tap into the two dimensions identified by the dual foundations framework, none provide a thorough operationalization of the dual foundations’ explanation for these dimensions.

Some measures of ideology resemble the two dimensions but do not measure enduring values dimensions. For example, ideology is often measured by attitudes about current affairs and policy issues, as in scales of economic and social conservatism (e.g., Everett, 2013). While attitudes about specific issues and policies are an important part of ideology (Brandt et al., 2019; Federico, 2019; Johnston and Ollerenshaw, 2020), attitudes concern particular issues and are likely to be culturally specific, making them different to values. By values, we mean ideas regarding general principles and collective goals that transcend attitudes toward particular issues (D’Andrade, 2008; Schwartz, 2012; Bergh and Sidanius, 2021). It is therefore not straightforward to infer enduring value dimensions purely from context-specific attitudes like those measured by scales of economic and social conservatism (Carmines and D’Amico, 2015; Federico et al., 2021).

Of contemporary scales that aim to measure values, three options seem promising. First, SDO and RWA are commonly paired together as a two-dimensional scale (Duckitt, 2001). This pairing is post-hoc, however, with each having independent origins in different research traditions (Altemeyer, 1981; Pratto et al., 1994). Also, rather than values regarding inequality *per se*, SDO ostensibly measures preferences for dominance relations between discrete social groups (Pratto et al., 1994). SDO is thus only incidentally useful for measuring broader preferences for competition and inequality, with which group dominance has only partial conceptual overlap (Kugler et al., 2010). RWA, meanwhile, ties authoritarianism to the political right. This is different from social control as conceived by the dual foundations framework: a value orientation that could theoretically be exhibited by people from any location on the “pro- versus anti-inequality” spectrum (Claessens et al., 2020). Furthermore, RWA relies on issues, words, and phrases that signal political commitments within a particular Western context, such as “God’s laws about abortion, pornography, and marriage must be strictly followed before it is too late” (Duckitt et al., 2010). Concerns thus exist in the political psychology literature that RWA conflates authoritarian values with attitudes toward right-wing social issues salient in particular cultural contexts (e.g., the United States and other Western democracies) (Van Hiel et al., 2007; Malka et al., 2017). This makes it problematic to use RWA to predict and explain social conservatism. Therefore, while we adopt recent versions of SDO and RWA in our analyses below because they are some of the most widely used scales of ideology’s two dimensions, we should remember they do not perfectly measure the dimensions theorized by the dual foundations framework.

A second potentially useful way of measuring political values is to examine them in a context other than country-level politics. This is done by the recent Nurturant Parent and Strict Father scales (Feinberg et al., 2020) that operationalize Lakoff (2016) moral politics thesis and which, in turn, builds on a long history of using family (and parenting) values as means to understand ideology (Fried, 1967, p. 83; Stenner, 2005). This research shows that values about family life comprise a two-dimensional structure that appears to mirror the two dimensions of national politics (Feinberg et al., 2020). These dimensions of family values relate to other two-dimensional measures in the expected ways, with Nurturant Parent correlating more strongly with SDO and Strict Father more strongly with RWA. Nevertheless, while the focus on family values represents an interesting context in which to study the dual foundations, it makes this scale more contextually specific than the dual foundations framework itself. We therefore view the Nurturant Parent and Strict Father dimensions as a useful extension of work on the two-dimensional structure of values, but do not see them as a full operationalization of the framework.

Third, Schwartz values offer another alternative that have been used to study political ideology (Schwartz et al., 2014; Caprara et al., 2017). Many of Schwartz’s measures of personal values seem to reflect the two dimensions. For example, Schwartz et al. (2012) Benevolence, Universalism, and Achievement values appear to track the inequality dimension, while Conformity, Tradition, and Self-Direction values appear to track the social control dimension. Nevertheless, the derivation of these Schwartz values is themselves somewhat theoretically unclear. Although Schwartz’s early work (Schwartz and Bilsky, 1987; Schwartz, 1992) does indicate that theorizing about trade-offs played some role in the development of these value dimensions, this work is not based on any explicit evolutionary approach. Later accounts make only tentative links between the value dimensions and “demands of human nature and requirements of societal functioning” (Schwartz, 2012, p. 15). Thus the number and content of value dimensions seems determined by exploratory processes of iterative testing and refinement rather than commitment to a first-principles evolutionary account. Moreover, Schwartz’s personal values are different to the political values expressed in scales like SDO and RWA. For example, RWA’s tripartite structure—upholding norms, submitting to leaders, and punishing deviance—is not reflected in Schwartz’s personal conservation values. Therefore, while existing scales may provide interesting data for the dual foundations approach, to properly close the gaps between current theory of political values and its operationalization, we need a new theory-driven scale of political values.

Here, we begin to address these concerns with the creation of the Dual Foundations Scale (DFS). We designed the DFS to operationalize the two trade-offs that the dual foundations framework identifies as underlying prior scales of the two dimensions of ideology. The Inequality dimension asks about preferences toward some people having more or fewer resources than others, and toward sharing resources more or less evenly with others. The DFS’s Social Control dimension asks about preferences toward following group rules, how harshly people should be punished for breaking group rules, and how rigidly people should follow group leaders. This means the DFS operationalizes ideology in a way that is both theoretically coherent and avoids a “dog-whistle” approach whereby particular phrases (e.g., “equality,” “competition,” “law and order”) or topics (e.g., religion, civil rights, gay, and women’s liberation) are given that prime self-placement within a specific cultural milieu.

The DFS extends existing scales in useful ways because it operationalizes ideology with a specific and unified theoretical approach derived from evolutionary theory (Claessens et al., 2020). First, DFS Inequality transcends SDO’s focus on dominance relations between discrete social groups and instead measures more general values about social inequality. DFS Inequality thus elicits preferences about some people having more resources than others without invoking group differences. The Inequality dimension also follows the evolutionary framework’s argument that inequality is tied to competitive rather than cooperative resource distribution. DFS Inequality thus also measures preferences toward the sharing of resources, again without invoking group differences. Second, DFS Social Control is not cast in the colors of either *right-wing* or *left-wing authoritarianism* (Conway et al., 2018; Nilsson and Jost, 2020). While DFS Social Control mirrors the tripartite structure of authoritarianism scales, it does so in a manner supported by independent research on the importance of social norms, sanctioning of deviance, and leadership for human collective activities (Pietraszewski, 2020).

The dual foundations framework predicts that the trade-offs are inherent to social groups of all sizes. The two dimensions of politics should thus exist across various levels of social organization. By employing items suitable to any named social group, the DFS enables analyses of political ideology across levels of social organization within the same society. For example, when conducting a study in the USA, one item from the DFS can read:

“When some *Americans* get a lot of resources, they share all of these resources with other *Americans*.”

The participant responds on a continuum from very bad to very good. This same item can then also be adapted to “family-level” politics simply by changing the names:

“When some *family members* get a lot of resources, they share all of these resources with other *family members*.”

While political psychologists do most frequently study “nation-level” politics, as we have seen with the Nurturant Parent and Strict Father scales, there is interest in studying the two dimensions of political values in contexts other than nation-level politics. The DFS’s ability to compare attitudes between different levels of politics thereby promises to provide insight into the degree of consistency and variation within individuals’ ideologies across different contexts.

This functionality means we also expect the Dual Foundations Scale will be a good candidate for future cross-cultural work. This follows from the dual foundations framework which the DFS operationalizes, which predicts that the two dimensions of politics reflect trade-offs that are inherent to human social life and, hence, ubiquitous across societies. By operationalizing the dual foundations framework’s two trade-offs, the DFS makes few assumptions about cultural context. Furthermore, because the DFS can be adapted to a new context by simply inserting the name of a social group, this allows it to be easily modified to suit different countries. It also permits application of the scale to contexts other than a nation-state, such as ‘Europeans” or “Punjabis.” This technique even enables application of the DFS to contexts where political life continues largely outside centralized institutions. For example, its items can be adapted to “members of village *x*” or “speakers of *y* language.” The DFS thus potentially allows researchers to pose the same questions about political values in settings both traditional to political psychology (industrialized and centralized nation-states) and more familiar to anthropologists (non-industrialized, industrializing, and economically peripheral communities), without reference to dog-whistle issues. In all, the DFS items provide flexibility alongside enough specificity that they remain concrete descriptions of the two trade-offs hypothesized to underly political ideology’s two dimensions.

This tactic of writing items applicable to multiple social groups, either within one society or across many, is influenced by Stellmacher and Petzel (2005) Group Authoritarianism scale. The DFS extends the Group Authoritarianism scale’s approach to include both Social Control (i.e., authoritarianism) and Inequality dimensions, providing an integrated and theory-driven two-dimensional scale and allowing assessment of both dimensions at once. Furthermore, at only 10 items, the DFS is shorter than Group Authoritarianism, making it versatile and permitting its inclusion in longer questionnaires. Stellmacher and Petzel (2005, p. 269) themselves recommend using simplified versions of their scale items in future work, but researchers adopting different simplifications would create measurement inconsistencies. The DFS constitutes a measurement instrument that is both simple and standardized.

Here, across two studies, we use a suite of pre-registered tests to validate the Dual Foundations Scale among an online sample of Americans. We chose an American sample because political ideology is well studied in this population. This allows validation of the new DFS in a context where the structure of political ideology can be predicted with certainty and where its properties can be compared against other scales already known to perform well. Validation takes two broad forms. First, tests of internal validity assess whether the DFS can effectively measure the two dimensions in the way expected by the dual foundations framework. Second, tests of external validity assess the DFS’s convergent and discriminant validity—that is, that the DFS predicts things that the dual foundations framework expects it to predict (convergent validity) and does not predict things that the dual foundations framework does not expect it to predict (discriminant validity).

## 2. Study 1

### 2.1. Study 1 introduction

In Study 1, we first refine the Dual Foundations Scale through the exclusion of items. This is a proper part of the scale creation process (Clark and Watson, 1995) and is included in our pre-registration document. Refinement of the DFS creates a succinct 10-item scale to measure the two dimensions outlined by the dual foundations framework.

Our first test of the new scale employs confirmatory factor analysis (CFA) to assess its internal validity. CFA is appropriate because, as the name suggests, it is used to test theory-derived predictions about the factor structure of observed variables (Suhr, 2006; Widaman, 2012). We test whether the DFS items’ covariance structure reflects the specific two-dimensional structure predicted by the dual foundations framework. We then test for three types of measurement invariance: configural, metric, and scalar. We aim to show that differences in participants’ scores reflect differences in the constructs rather than differences in their measurement (Putnick and Bornstein, 2016).

We then move on to explore relationships between the DFS dimensions themselves, using structural equation models (SEMs). Of key interest is the relationship between participants’ DFS scores across two levels of social organization: nation and family. We examine the nation level due to its prevalence in the politics literature and include the family level as a comparison given the long history in psychology of links between politics and family and parenting values (Stenner, 2005; Barker and Tinnick, 2006). Theories of political ideology expect consistency across these two levels, either because people have stable dispositions that are expressed across both (e.g., Duckitt and Sibley, 2009) or because opinions about nation-level politics derive from opinions about family-level politics (e.g., Lakoff, 2016). We therefore test whether DFS Nation Inequality is predicted by Family Inequality (more than Family Social Control), and DFS Nation Social Control is predicted by Family Social Control (more than Family Inequality). This would demonstrate convergent validity in that, across the two levels of social organization, DFS Inequality and Social Control measure constructs that are related, and discriminant validity in that the two dimensions each measure their own construct and not the other.

To test external validity, we next test the DFS’s relationships with existing politics scales. First, we test whether the two DFS dimensions predict right-wing politics in general. In Western nations, research frequently characterizes the political right with support for both inequality and social control (Azevedo et al., 2019). We therefore predict that both DFS Inequality and Social Control will independently predict right-wing self-placement on a unidimensional scale. Second, since SDO and RWA are two of the most widely researched measures of the two dimensions, we test whether the DFS dimensions show convergent and discriminant validity in relation to SDO and RWA. Third, we test relationships between the DFS and family values. We test whether the DFS dimensions show convergent and discriminant validity in relation to the two dimensions of Nurturant Parent and Strict Father (Feinberg et al., 2020).

Schwartz’s values are another promising measure of the two dimensions of ideology, so we test the DFS’s external validity in relation to values from Schwartz et al. (2012) refined Portrait Values Questionnaire (PVQ). We expect DFS Inequality and Social Control to show convergent and discriminant validity in relation to eight relevant values. For the Inequality dimension, these are: Benevolence-Caring, Benevolence-Dependability, Universalism-Concern, and Achievement. For the Social Control dimension: Tradition, Conformity-Rules, Self-Direction-Action, and Self-Direction-Thought.

Aside from psychometric scales, the dual foundations framework’s focus on trade-offs opens another avenue for measuring political ideology with incentivized tasks, a suite of tools that is gaining popularity to measure social behavior (Pisor et al., 2020). Since the dimensions are grounded in trade-offs inherent to group-living, predictions have been made about each dimension’s ties to preferences in incentivized tasks (Claessens et al., 2020; Fischer et al., 2021). Recent work shows that each ideological dimension is associated with particular behaviors in different tasks. Anti-equality attitudes (as measured by SDO) correlate with self-serving (rather than pro-social) choices in Dictator Games, Ultimatum Games, and Public Goods Games (Claessens et al., 2021). Socially controlling attitudes (measured by RWA), meanwhile, correlate with rule following behaviors in incentivized tasks (Fischer et al., 2021). To establish relationships between the DFS and behavioral measures, we aim to show that the two dimensions show convergent and discriminant validity in relation to a Dictator Game and a Rule Following task.

We pre-registered our hypotheses with the Open Science Framework (OSF) before collecting the data (doi: 10.17605/OSF.IO/ZYN9W).

### 2.2. Study 1 materials and methods

#### 2.2.1. Scale development

The scale development process took several months. We wrote 24 items describing situations outlined by the dual foundations framework. We gained feedback on proposed items from colleagues and laypeople and ensured a balance between items worded “pro-trait” and “con-trait.” Before running Study 1, we conducted two pilot tests of *N* = 50. After each pilot, we assessed how well individual items worked and reviewed written participant feedback, which we used to alter wordings.

#### 2.2.2. Participants and procedure

Given the difficulty of *a priori* estimating sample size for SEMs (Wolf et al., 2013), we based recruitment on our prior work using SEMs to regress latent variables of political ideology with incentivized behavioral tasks (Claessens, 2021). In August 2020, we recruited 501 American participants from Prolific^1^ to complete a questionnaire hosted by Qualtrics.^2^ Participants answered a battery of scales and tasks with a mean completion time of 35.5 min (*SD* = 24.4). All were compensated US$7 for participation, plus bonus payments based on decisions in the incentivized tasks (total stakes US$2.80). Twelve participants did not complete the survey or failed an attention check and were excluded, leaving 489 (see Supplementary material).

#### 2.2.3. Materials

All regressions described below control for five standard covariates: the effects of the participant being a man, their age, religion, education, and income. In response to a tick-box question, 228 identified as male (46.6%), while 251 identified as female (51.3%) and 10 as neither male nor female (2%). Mean self-reported age was 30.9 (*SD* = 11.7). Religion was measured with a binary variable that asked whether participants identified with a religious or spiritual group: 290 (59.3%) said no and 199 (40.7%) said yes. Education was measured on a seven-point ordinal scale with a median of 4 (college with associate’s degree). Income was a self-reported estimate of yearly household income before tax, measured on a 13-point ordinal scale with a median of 6 (US$50,000–59,000).

Each participant completed the DFS twice: once about America and once about their family. We randomized the order of these versions. DFS items were presented in random order, as is true for all scales. Participants responded to the DFS items with a slider displaying values from 0 to 100. After reverse-coding appropriate items, a score of 100 indicated a participant thought inequality or social control was “very good,” 50 indicated “neither good nor bad,” and 0 indicated “very bad.” We report DFS means and reliability statistics in the results.

Participants responded to a single self-report measure of “left versus right” political orientation on a 0–100 scale. 0 was “left,” 50 was labeled “center,” and 100 was “right.” We told them that the Democratic Party was described as more to the left, and the Republican Party as more to the right. The mean score was 31.49 (*SD* = 26.59).

To measure SDO, we used Ho et al. (2015) 16-item scale. To measure RWA, we used Duckitt et al. (2010) 18-item scale. Scale items are provided in Supplementary material, as is true for the scales described below. After reverse-coding appropriate items, a score of 100 indicated strong agreement, 50 indicated neither agreement nor disagreement, and 0 indicated strong disagreement. Mean SDO was 23.23 (*SD* = 17.79; McDonald’s total omega reliability = 0.93, hereafter *ω*) and mean RWA was 33.26 (*SD* = 20.68; *ω* = 0.94).

We adopted the Moral Politics Scale’s two dimensions of Nurturant Parent (15 items) and Strict Father (14 items) (Feinberg et al., 2020). After reverse-coding the appropriate items, a score of 100 indicated strong agreement, 50 indicated neither agreement nor disagreement, and 0 indicated strong disagreement. We then reversed Nurturant Parent (hereafter Nurturant Parent^R^) so it should correlate positively, rather than negatively, with DFS Inequality. Mean Nurturant Parent^R^ was 20.87 (*SD* = 12.1; *ω* = 0.85) and Strict Father was 50.07 (*SD* = 16.84; *ω* = 0.90).

Schwartz et al. (2012) refined PVQ maps 19 value dimensions by asking participants to rate how similar descriptions of a hypothetical person are to themselves. We did not gender this hypothetical person. Participants rated how much the person was like themselves on a scale from 0 (“strongly disagree”) to 100 (“strongly agree”); 50 indicated neither agreement nor disagreement. We made pre-registered predictions about eight of these values. We reverse-coded five of the eight values so that higher scores should always connote more conservative opinions. Four “inequality-type” values were: Benevolence-Caring^Reversed^ (mean = 14.82, *SD* = 13.79, *ω* = 0.84), Benevolence-Dependability^Reversed^ (mean = 26.04, *SD* = 18.11, *ω* = 0.43), Universalism-Concern^Reversed^ (mean = 17.66, *SD* = 15.77, *ω* = 0.77), and Achievement (mean = 64.71, *SD* = 19.34, *ω* = 0.69). Four more “social control-type” values were: Tradition (mean = 42.99, *SD* = 27.85, *ω* = 0.90), Conformity-Rules (mean = 62.5, *SD* = 24.35, *ω* = 0.81), Self-Direction-Thought^Reversed^ (mean = 18.34, SD = 13.48, *ω* = 0.66), and Self-Direction-Action^Reversed^ (mean = 21.55, SD = 12.85, *ω* = 0.65). We used mean scores because the PVQ values rely on two to three items each.

Following from the prior work discussed above that links cooperative behavior and political ideology, we used a Dictator Game to measure cooperative preferences. Participants could split US$1 between themselves and another anonymous participant. It thus measures an individual’s willingness to pay a cost to aid another (Engel, 2011; House et al., 2020). Scores could range from 0 to 100. The average amount participants allocated to themselves, which we call Dictator Game Keepings, was 58 cents (*SD* = 0.21).

Following previous work exploring rule following and ideology, we used a Rule Following task to measure social control preferences. Each participant had to move 30 balls into two buckets with different payoff rates. For instance, for every ball put into bucket A they would earn US$0.06 but only US$0.03 for bucket B. The participant would be told the rule is to put the balls into bucket B. This creates a conflict between following an arbitrary rule and self-interest (Kimbrough and Vostroknutov, 2018). Participants were sorted into one of two ball tasks, where either bucket A was the self-interest-maximizing and rule-breaking choice, or bucket B was the self-interest-maximizing and rule-breaking choice. The Rule Following measure was the number of balls put into the non-self-interest-maximizing bucket. The average participant put 20.58 (*SD* = 11.93) balls into this rule-following bucket.

#### 2.2.4. Data analysis

Analysis was conducted using R (4.1.0) (R Core Team, 2020). The *psych* package (Revelle, 2019) was used for calculating McDonald’s total omega reliability (*ω*). The omega is a better measure of scale reliability than Cronbach’s alpha, which is a poor measure (Dunn et al., 2014; Peters, 2014; Trizano-Hermosilla and Alvarado, 2016). Factor analyses and structural equation modeling used the *lavaan* package (Rosseel, 2012). We estimated CFAs using diagonally weighted least squares (DWLS). We used SEMs to perform regressions between latent variables, controlling for the demographic factors outlined above. We conduct SEMs with the DFS dimensions as both independent and dependent variables, which allows us to comprehensively test whether the DFS dimensions show the expected correlations with other two-dimensional measures of politics, holding both the other DFS dimension or the alternate measure’s other dimension constant. We present the regression results in the form of standardized regression coefficients—these are measures of effect size (Nieminen et al., 2013). The SEMs utilize maximum likelihood (ML) estimation because many models failed to converge using DWLS. We found no qualitative differences in regression estimates for those models for which we could perform both ML and DWLS estimation. Analysis code and de-identified data are available to ensure the statistical reproducibility of all reported figures, tables, and results (doi: 10.17605/OSF.IO/SYB9H).

#### 2.2.5. Ethics statement

We gained approval from the University of Auckland Human Participants Ethics Committee on 22/06/2020 (ref. 024358).

### 2.3. Study 1 results

#### 2.3.1. Refining the scale

We began by selecting the Dual Foundations Scale’s items. We favored items with higher inter-item correlations, while also keeping balanced the number of items covering each of the sub-dimensions and the number of items that were worded pro-trait and con-trait. During scale development, we had considered including a third topic inside the Inequality dimension that was about inequality in power. We debated whether this topic contained too much conceptual overlap with Social Control. Due to our pre-existing concerns of content overlap and this topic showing relatively lower inter-item correlations than the other items, we dropped these items in this first stage of analysis. This resulted in a 10-item scale with four items for DFS Inequality and six for DFS Social Control. The full list of item wordings (for both DFS Nation and Family), with inter-item correlations (Spearman’s rank) before and after dropping items, is available in Supplementary material. The 10-item DFS obtained good reliability: Nation Inequality *ω* = 0.73, Nation Social Control *ω* = 0.78, Family Inequality *ω* = 0.72, Family Social Control *ω* = 0.79. Mean responses were: 26.84 out of 100 for Nation Inequality (*SD* = 16.63), 53.04 for Nation Social Control (*SD* = 15.23), 28.49 for Family Inequality (SD = 14.81), and 46.42 for Family Social Control (SD = 15.48).

#### 2.3.2. Does the DFS produce two clear factors?

CFAs demonstrate good fit of our data to the hypothesized two-dimension model. All items load positively and significantly onto the expected dimensions (Table 2). We obtained good fit by Hu and Bentler (1999) recommendations: Comparative Fit Index (CFI) scores above 0.950, Root Mean Square Error of Approximation (RMSEA) values below 0.060, and Standardized Root Mean Square Residual (SRMR) values below 0.080. Table 3 provides fit indices for the hypothesized two-dimension models for DFS Nation and Family and shows that each clearly outperforms two alternatives: a model with a single dimension and a model with two dimensions that represent the items’ pro- versus con-trait wordings. Table 3 also demonstrates that the two-dimensional models of DFS Nation and Family worked well compared with the two-dimensional models of SDO-RWA and Nurturant Parent^R^-Strict Father. This supports the theoretical motivations for creating the DFS scale: values about inequality and competition cluster together into a coherent factor, as do values about submission to group norms, punishment, and authoritative leadership.

**Table 2:** Standardized item loadings for DFS Nation’s two dimensions and DFS Family’s two dimensions.

Version	Dimension	Wording	Loading	CI (95%)	Z score	*p-*value
Nation	Inequality	When some Americans get a lot of resources, they do not share any of their resources with other Americans.	0.706	0.591, 0.820	12.064	<0.001
		When some Americans get a lot of resources, they share all of these resources with other Americans.	0.485	0.386, 0.585	9.565	<0.001
		Some Americans do not get the resources that other Americans have, which means that some Americans have less than others.	0.830	0.699, 0.962	12.377	<0.001
		Some Americans try to make sure that no Americans get fewer resources than other Americans.	0.474	0.386, 0.562	10.570	<0.001
	Social control	All Americans have to follow all of the USA’s rules all of the time.	0.720	0.625, 0.815	14.907	<0.001
		An American does not follow some of the USA’s rules because they do not agree with them.	0.748	0.642, 0.854	13.841	<0.001
		The USA punishes an American very harshly, because they have repeatedly broken the USA’s rules.	0.438	0.355, 0.522	10.265	<0.001
		The USA does not punish an American after they have broken a US rule which you do not think is important.	0.449	0.368, 0.531	10.817	<0.001
		Some Americans do not agree with a decision made by the USA’s leaders, but they have to follow the leaders’ decision anyway.	0.589	0.498, 0.680	12.653	<0.001
		An American does not follow the decision of the USA’s leaders, because they do not agree with the leaders’ decision.	0.672	0.570, 0.774	12.907	<0.001
Family	Inequality	When some family members get a lot of resources, they do not share any of their resources with other family members.	0.724	0.597, 0.852	11.120	<0.001
		When some family members get a lot of resources, they share all of these resources with other family members.	0.603	0.488, 0.718	10.279	<0.001
		Some of your family members do not get the resources that other family members have, which means that some family members have less than others.	0.626	0.508, 0.743	10.442	<0.001
		Some members of your family try to make sure that no family members get fewer resources than other family members.	0.529	0.431, 0.627	10.590	<0.001
	Social control	All members of your family have to follow all of the family’s rules all of the time.	0.693	0.602, 0.784	14.871	<0.001
		A family member does not follow some of your family’s rules because they do not agree with them.	0.722	0.619, 0.825	13.776	<0.001
		Your family punishes a family member very harshly, because they have repeatedly broken your family’s rules.	0.490	0.410, 0.569	12.043	<0.001
		Your family does not punish a family member after they have broken a family rule which you do not think is important.	0.444	0.365, 0.523	10.973	<0.001
		Some members of your family do not agree with a decision made by the family’s leaders, but they have to follow the leaders’ decision anyway.	0.659	0.568, 0.751	14.100	<0.001
		A family member does not follow the decision of your family’s leaders, because they do not agree with the leaders’ decision.	0.703	0.601, 0.806	13.435	<0.001

**Table 3:** Fit indices for models using the DFS data, as well as SDO-RWA and Nurturant Parent (reversed)-Strict Father.

Model	CFI	RMSEA	SRMR
DFS Nation: Inequality and social control dimensions	0.968	0.045	0.058
DFS Nation: Single dimension	0.694	0.139	0.133
DFS Nation: Pro-trait and con-trait dimensions	0.695	0.141	0.132
DFS Family: Inequality and social control dimensions	0.987	0.029	0.051
DFS Family: Single dimension	0.690	0.139	0.136
DFS Family: Pro-trait and con-trait dimensions	0.691	0.141	0.135
SDO and RWA: Two dimensions	0.991	0.034	0.065
Nurturant Parent (r) and Strict Father: Two dimensions	0.968	0.042	0.064

CFI is the Comparative Fit Index, RMSEA is the Root Mean Square Error of Approximation, and SRMR is the Standardized Root Mean Residual.

#### 2.3.3. Does the DFS demonstrate measurement invariance between groups?

We find support for our pre-registered predictions about measurement invariance. Table 4 presents invariance test results for DFS Nation and Family across three binary variables: sex, age, and ethnicity. To look at invariance with regards to sex, we focus on those that identified as either male or female. To create the binary age variable, we performed a median split where median age is 27 years. The 27-and-under group contained 247 people (mean = 22.2, *SD* = 2.88), while over-27 group contained 242 (mean = 39.2, *SD* = 10.6). For the binary ethnicity variable, we grouped together those respondents who identified only as white (*N* = 311) and all those identifying with any non-white ethnicity (*N* = 178). The results show that both Nation and Family versions of DFS were invariant across these groups, since the metric invariance models all differ by less than 0.01 compared to the configural invariance models, and the scalar invariance models all differ by less than 0.01 to the metric invariance models (Fischer and Karl, 2019). This supports our hypothesis that the DFS measures the same constructs, in the same way, across different groups within our sample.

**Table 4:** Fit indices for DFS models, with equality constraints for testing configural, metric, and scalar invariance.

Model	Invariance type	CFI	RMSEA	SRMR
Model 1 (*N* = 479)				
DFS Nation split by sex	Configural	0.983	0.033	0.060
DFS Nation split by sex	Metric	0.982	0.032	0.062
DFS Nation split by sex	Scalar	0.986	0.027	0.063
Model 2 (*N* = 489)				
DFS Nation split by age	Configural	0.980	0.036	0.061
DFS Nation split by age	Metric	0.980	0.034	0.063
DFS Nation split by age	Scalar	0.981	0.032	0.065
Model 3 (*N* = 489)				
DFS Nation split by ethnicity	Configural	0.981	0.037	0.060
DFS Nation split by ethnicity	Metric	0.977	0.038	0.064
DFS Nation split by ethnicity	Scalar	0.972	0.040	0.068
Model 4 (*N* = 479)				
DFS Family split by sex	Configural	1.000	0.000	0.054
DFS Family split by sex	Metric	1.000	0.000	0.056
DFS Family split by sex	Scalar	1.000	0.000	0.058
Model 5 (*N* = 489)				
DFS Family split by age	Configural	0.994	0.020	0.058
DFS Family split by age	Metric	0.988	0.026	0.063
DFS Family split by age	Scalar	0.982	0.031	0.067
Model 6 (*N* = 489)				
DFS Family split by ethnicity	Configural	0.995	0.018	0.057
DFS Family split by ethnicity	Metric	0.995	0.018	0.060
DFS Family split by ethnicity	Scalar	0.993	0.019	0.063

CFI is the Comparative Fit Index, RMSEA is the Root Mean Square Error of Approximation, and SRMR is the Standardized Root Mean Residual.

#### 2.3.4. Does the DFS show convergent and discriminant validity between family and nation?

**Figure 1:**
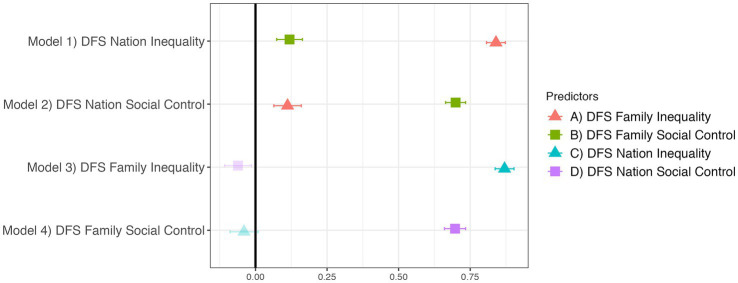
DFS Family predicts DFS Nation and vice versa, across corresponding dimensions. The outcome variable of each model is given on the y-axis, with the predictors’ effects plotted on the x-axis. Translucent points indicate *p* ≥ 0.05. Regression estimates are standardized betas with standard errors. All four models control for demographic covariates and religion.

Figure 1 supports our pre-registered hypotheses that DFS Nation Inequality would be more strongly predicted by DFS Family Inequality than Family Social Control, and that Nation Social Control would be more strongly predicted by Family Social Control than Family Inequality. Figure 1 also presents the results of two non-preregistered exploratory analyses, which indicate that DFS Nation Inequality but not Nation Social Control is a significant predictor of Family Inequality, and that DFS Nation Social Control but not Nation Inequality is a significant predictor of Family Social Control. These results show each dimension is strongly and consistently related to itself across the two levels of social organization, supporting prior arguments for consistency in individuals’ opinions regarding the two dimensions (Duckitt and Sibley, 2009). Furthermore, the different dimensions do not strongly predict each other across these levels of social organization, indicating that they measure independent constructs.

#### 2.3.5. How do the two dimensions of ideology relate to each other?

In exploratory analyses presented in Supplementary material, we investigate relationships between each of our two-dimensional measures of ideology. As expected, SDO and RWA relate positively to each other. We also find a positive relationship between Nurturant Parent^R^ and Strict Father. Interestingly, while there is a relatively weak positive relationship between DFS Nation Inequality and Social Control, Family Inequality and Social Control are not reliably related to each other. This could support an argument made by some researchers that the strong positive relationship between SDO and RWA partly reflects content overlap due to RWA’s conflation of authoritarianism with general right-wing attitudes (Van Hiel et al., 2007; Malka et al., 2017). The two DFS dimensions may therefore be less strongly related to each other than SDO-RWA due to more clearly distinguishing support for social control from generic right-wing attitudes.

#### 2.3.6. Do both DFS dimensions predict right-wing politics?

**Figure 2:**
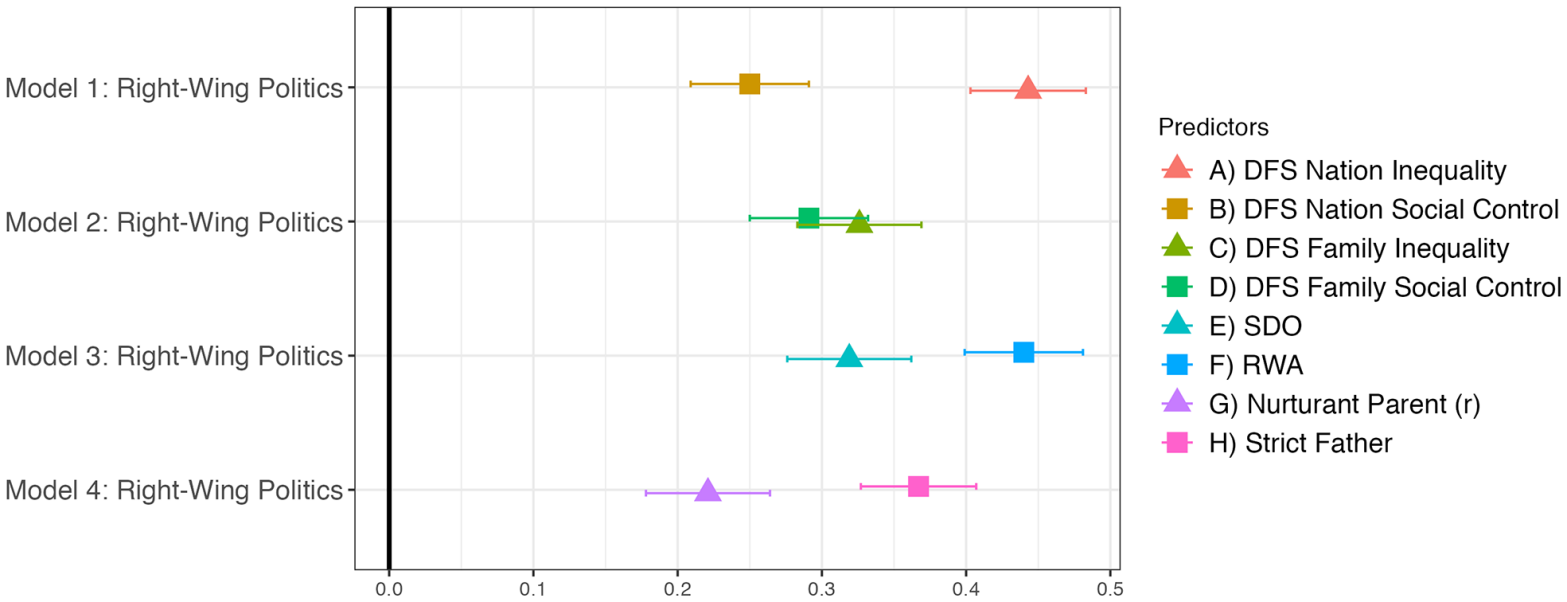
The DFS predicts right-wing politics as strongly as other measures of ideology. Translucent points indicate *p* ≥ 0.05. Regression estimates are standardized betas with standard errors. All four models control for demographic covariates and religion.

Figure 2 supports our pre-registered hypothesis that each of the DFS dimensions should predict right-wing politics. Inequality and Social Control (both Nation and Family) independently contribute to right-wing self-placement. Also in Figure 2, exploratory analyses show similar relationships between right-wing politics and SDO-RWA and Nurturant Parent^R^-Strict Father. Similarities in the effects of each scale’s two dimensions on right-wing self-placement further support our hypothesis that the DFS measures similar constructs to previous scales of political ideology.

#### 2.3.7. Does the DFS show convergent and discriminant validity in relation to SDO-RWA?

**Figure 3:**
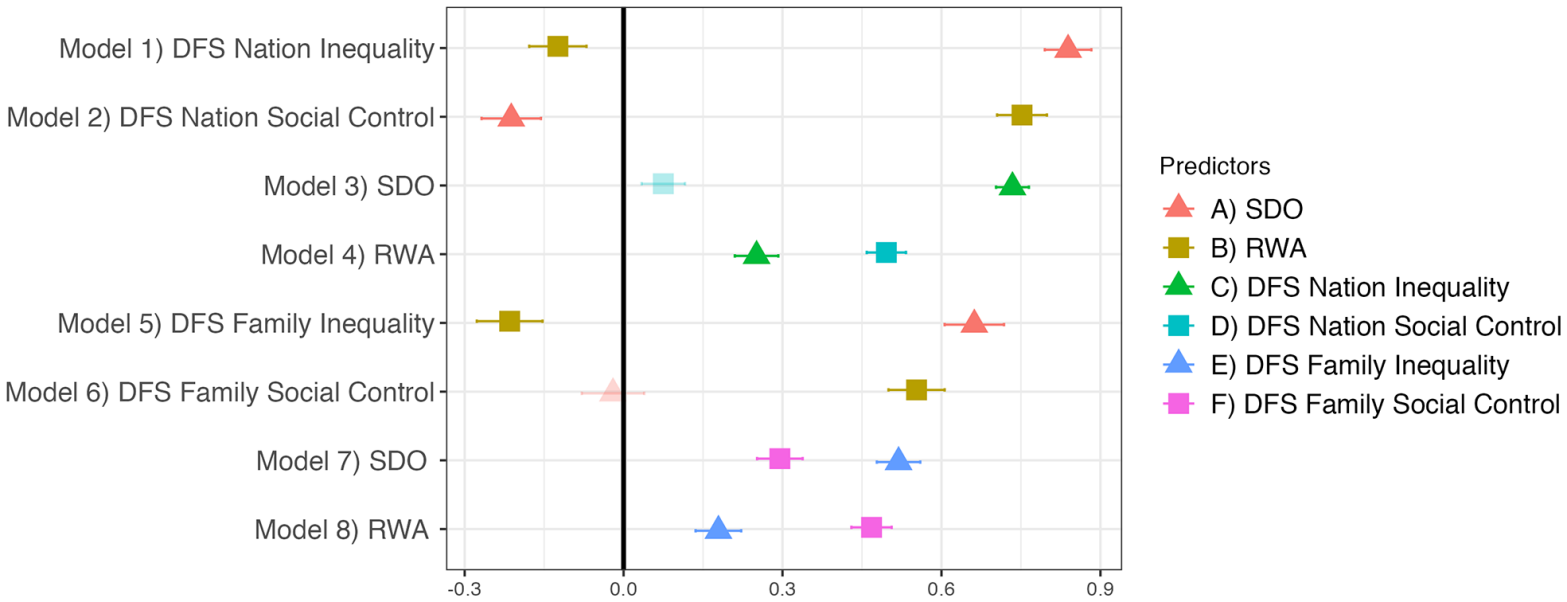
The DFS strongly relates to SDO and RWA. Translucent points indicate *p* ≥ 0.05. Regression estimates are standardized betas with standard errors. All eight models control for demographic covariates and religion.

Figure 3 provides justification for the DFS’s external validity against two of the most popular scales of political ideology. We support our pre-registered hypotheses that DFS Inequality should be more positively predicted by SDO than RWA, and DFS Social Control should be more positively predicted by RWA than SDO. Furthermore, Figure 3 supports our pre-registered hypotheses that SDO should be more positively predicted by DFS Inequality than Social Control, and RWA should be more positively predicted by DFS Social Control than Inequality. The strength and consistency of the effect sizes support our hypothesis that the DFS measures constructs which are highly similar to SDO-RWA.

#### 2.3.8. Does the DFS show convergent and discriminant validity in relation to Nurturant Parent^R^-Strict Father?

**Figure 4:**
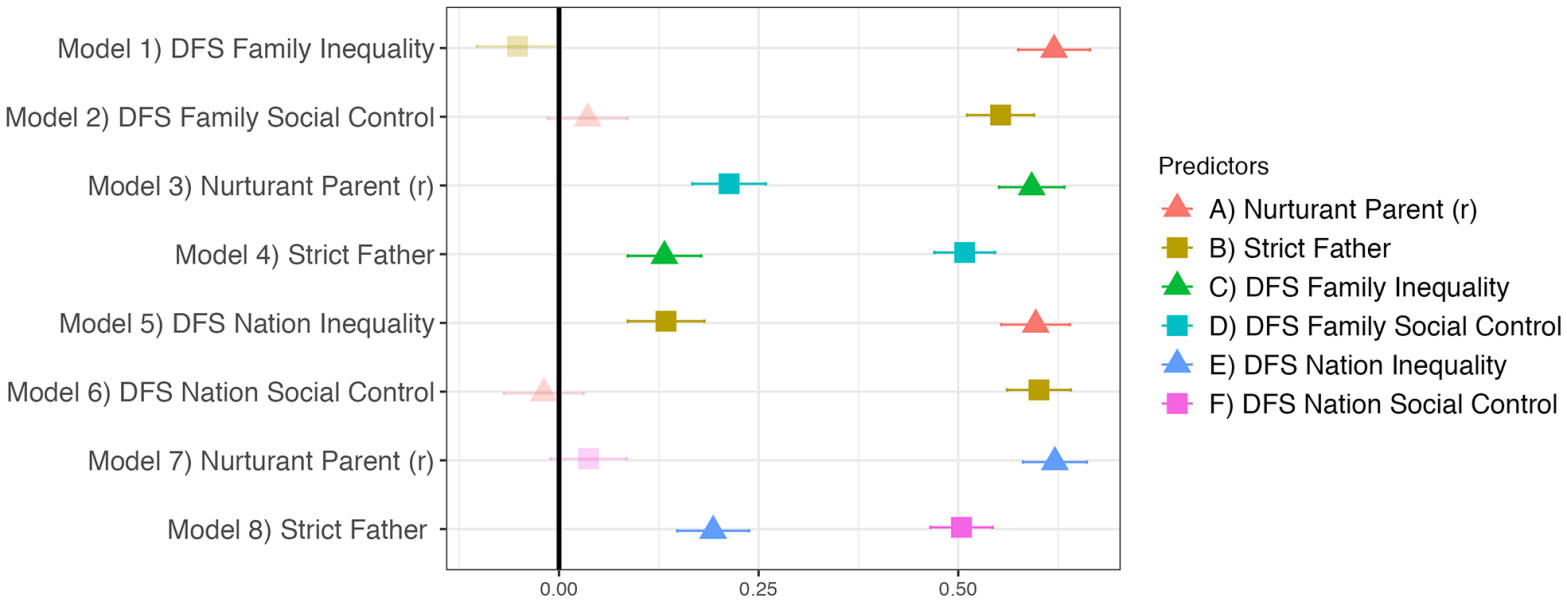
The DFS strongly relates to Nurturant Parent^R^ and Strict Father. Translucent points indicate *p* ≥ 0.05. Regression estimates are standardized betas with standard errors. All eight models control for demographic covariates and religion.

Figure 4 provides support for our pre-registered hypotheses that DFS Family Inequality should be more positively predicted by Nurturant Parent^R^ than Strict Father, that DFS Family Social Control should be more positively predicted by Strict Father than Nurturant Parent^R^, that Nurturant Parent^R^ should be more positively predicted by DFS Family Inequality than Social Control, and Strict Father should be more positively predicted by DFS Family Social Control than Inequality. Figure 4 also presents the results of four non-preregistered analyses, which replicate the same relationships using DFS Nation Inequality and Social Control. These results indicate that the DFS measures constructs that are reliably related to the Nurturant Parent-Strict Father dimensions.

#### 2.3.9. Does the DFS show convergent and discriminant validity in relation to Schwartz PVQ values?

We preregistered predictions about the external validity of the DFS in relation to eight Schwartz PVQ values. Before conducting these analyses, we checked whether the Schwartz values correlated with each other as expected. We found that only five of the values worked as intended: Benevolence-Caring, Benevolence-Dependability, Universalism-Concern, Tradition, and Conformity-Rules. Despite Achievement being designed to measure “the underlying motivation to be judged successful by others” which “motivate[s] people to compete,” and occupying the opposed segment to Benevolence-Caring in Schwartz’s value circle (Schwartz et al., 2012, pp. 669, 681), it correlated positively with Benevolence-Caring, Benevolence-Dependability, and Universalism-Concern. Likewise, despite Self-Direction’s opposition to Tradition in Schwartz’s value continuum, and indications that self-direction motivations should oppose conformity and social conservatism (Malka et al., 2014), neither Self-Direction-Thought nor -Action correlated negatively with Tradition or Conformity-Rules and in fact correlated positively with Benevolence-Caring, Benevolence-Dependability, and Universalism-Concern. These findings are presented in Supplementary material. Since these PVQ values do not seem to represent the constructs we intended, our pre-registered predictions regarding them are uninterpretable. We therefore do not include tests of these predictions here, though they are available in Supplementary material.

**Figure 5:**
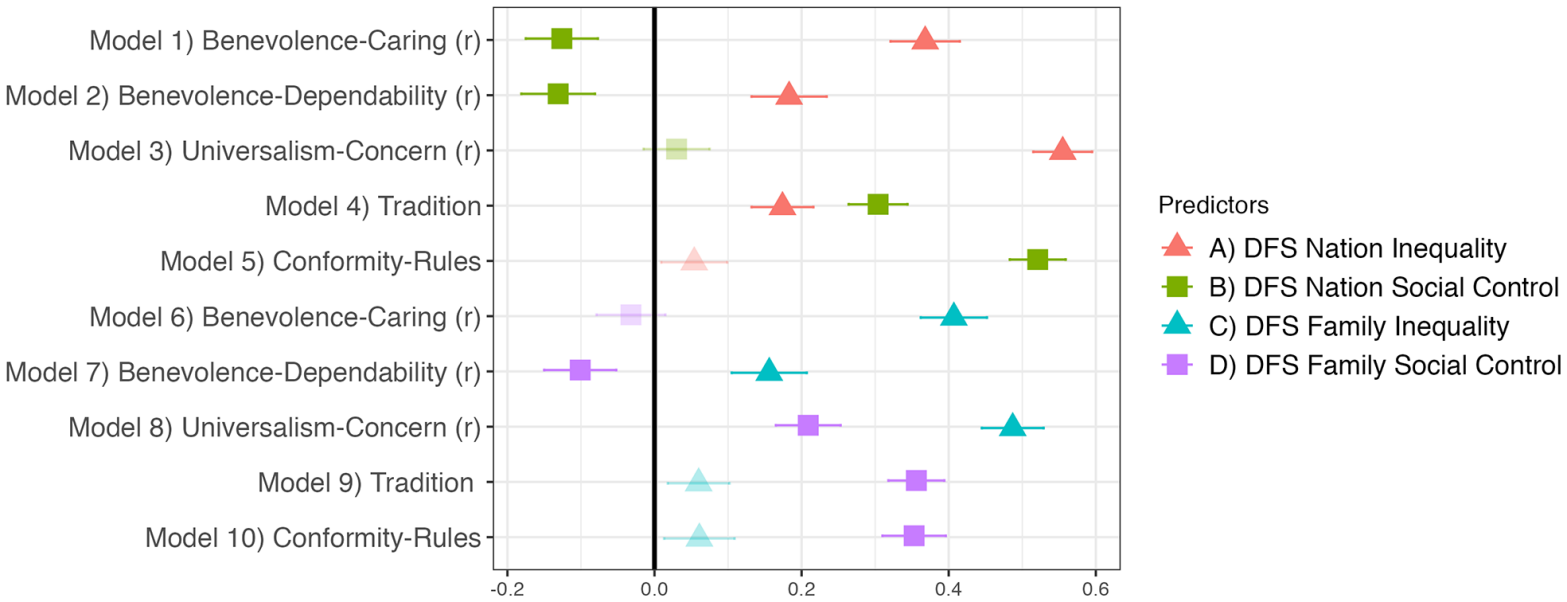
The DFS relates to select Schwartz PVQ values. Translucent points indicate *p* ≥ 0.05. Regression estimates are standardized betas with standard errors. All 10 models control for demographic covariates and religion.

Figure 5 demonstrates external validity of the DFS against the remaining Schwartz values. We find support for our hypotheses that DFS Inequality (more than Social Control) should positively predict values of Benevolence-Caring^R^, Benevolence-Dependability^R^, and Universalism-Concern^R^, and that DFS Social Control (more than Inequality) should positively predict the social control-type values of Tradition and Conformity-Rules. This pattern was identical for DFS Nation and Family. We also support most of our predictions about the predictive effects of Schwartz’s values on the DFS dimensions, as presented in Supplementary material. There, we examine the effects of each Schwartz value, controlling for the Schwartz values associated with the other dimension, on either DFS Inequality or Social Control. We find that each inequality-type Schwartz value predicts DFS Inequality independently of the social control-type values, and each social control-type Schwartz value predicts DFS Social Control independently of the inequality-type values. However, two Schwartz values occasionally also predict the unanticipated DFS dimension: DFS Social Control was sometimes predicted by Universalism-Concern^R^, while DFS Inequality was sometimes predicted by Tradition even more strongly than it was by values like Benevolence-Dependability. Do these unexpected patterns call into question the external validity of the DFS? We think not, because we find the same relationships between Universalism-Concern and both RWA and Strict Father, and between Tradition and both SDO and Nurturant Parent^R^ (further details in Supplementary material). Hence, rather than indicating a problem with the DFS, these findings suggest it may be problematic to use some specific Schwartz values as stand-ins for the two dimensions of ideology since some values relate to both dimensions of ideology, as measured by the DFS and other scales.

#### 2.3.10. Does the DFS show convergent and discriminant validity in relation to incentivized behavioral measures of the two dimensions?

**Figure 6:**
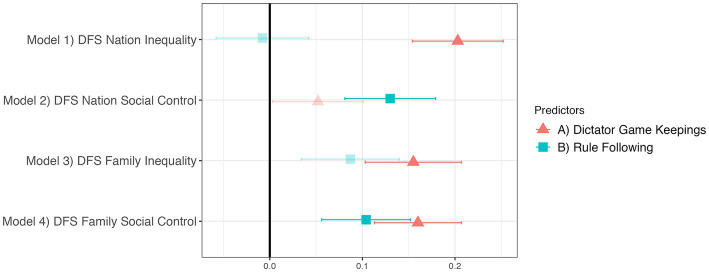
The DFS relates to Dictator Game Keepings and Rule Following. Translucent points indicate *p* ≥ 0.05. Regression estimates are standardized betas with standard errors. All four models control for demographic covariates, religion, and the other DFS dimension.

**Figure 7:**
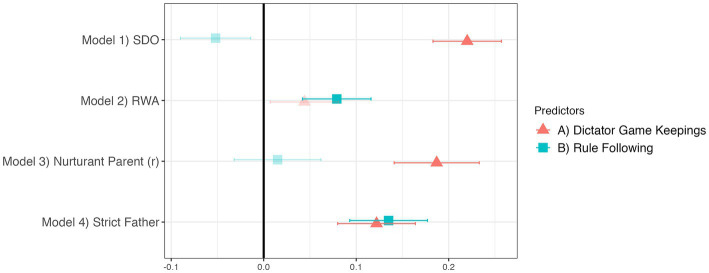
Other ideology scales relate to Dictator Game Keepings and Rule Following in a similar way. Translucent points indicate *p* ≥ 0.05. Regression estimates are standardized betas with standard errors. All four models control for demographic covariates, religion, and the other politics dimension.

Our predictions about the relationship of the DFS to incentivized tasks were partially supported. Importantly, these results are largely invariant to using either the DFS or alternative scales, indicating that the DFS measures ideology in relation to behavioral tasks at least as well as existing scales. This can be seen in Figures 6, 7, where the plotted regression results also control for the other dimension of ideology, which ensures we investigate the behavioral tasks’ relationships with the one specific dimension and not more general political preferences. Dictator Game Keepings independently predicts DFS Inequality (Nation and Family), SDO, and Nurturant Parent^R^. Rule Following independently predicts DFS Social Control (Nation and Family), RWA, and Strict Father. We also found an unexpected result: Dictator Game Keepings positively predicts DFS Family Social Control and Strict Father, at least as strongly as does Rule Following. Nevertheless, these results generally support the predictions of the dual foundations framework by demonstrating relationships between stated political values and behavior in incentivized tasks.

Further analysis of the behavioral tasks was complicated by the highly non-Gaussian distribution of responses. To model the effects of the DFS on Dictator Game Keepings we employed quantile regression, which is used where the assumptions of standard linear regression are broken. The results of these quantile regressions are presented in detail in Supplementary material. All four measures of the inequality dimension (DFS Nation, DFS Family, SDO, and Nurturant Parent^R^) positively predict Dictator Game Keepings in the 0.8 quantile, while SDO also predicts it in the 0.6 quantile. This seems due to the bunching of Dictator Game Keepings responses between 50 and 100, meaning that there is not enough variation for the predictor variables to have much effect below the most popular choice of 50 cents (itself chosen by 311 out of 489 participants, with only 24 participants choosing to keep less). To model the DFS’s effects on Rule Following we adopted a multilevel Bayesian approach. We find that only DFS Nation Social Control consistently predicts Rule Following, while the 95% credible intervals for the effects of DFS Family Social Control, RWA, and Strict Father on Rule Following each include negative as well as positive values. Detailed description of these results is in Supplementary material. Overall, we find qualified support for the dual foundations approach’s predictions of relationships between incentivized task preferences and political ideology, with the DFS at least as able to demonstrate these relationships as previous politics scales.

## 3. Study 2

### 3.1. Study 2 introduction

Study 2’s first aim is to examine the properties of the Dual Foundations Scale with new data. In Study 1, we used the same data to refine and test the scale. We therefore need to collect new data to ensure the DFS demonstrates the same properties when data is collected only for the final 10-item scale and without removing any further items. We assess this by examining CFA model fit scores and conducting the same measurement invariance tests as done in Study 1. We do not claim this constitutes a fully independent test of the DFS factor structure because Study 2’s participants are drawn from those that completed Study 1. However, recontacting the same participants does allow us to fulfill Study 2’s second aim, to examine the test–retest reliability of the DFS across an interval of 7 months. We first test for between-wave measurement invariance, and then examine correlations across waves. As previous research indicates SDO and RWA are relatively stable within individuals over time (Osborne and Sibley, 2020), we want to show participants’ DFS scores are also relatively stable.

Study 2’s second aim is to test a new behavioral measure of conformism. Recent research shows that RWA is related to a measure of participants’ reliance on social information in an incentivized perceptual estimation task (Claessens et al., 2021). This task, the BEAST (Berlin Estimate AdjuStment Task), measures how much participants’ rely on social information when they can win more money by giving more accurate answers (Molleman et al., 2019). We therefore expect reliance on social information to be more strongly related to DFS Social Control than Inequality (both Nation and Family).

We pre-registered our hypotheses with OSF before collecting the data (doi: 10.17605/OSF.IO/2NAFC).

### 3.2. Study 2 materials and methods

#### 3.2.1. Participants and procedure

We attempted to re-contact Study 1’s 489 participants via Prolific. Between March and April 2021, 289 responded and each was given US$3.50. Data collection was ended after a period of three consecutive working days without a new response. Ten participants did not complete the survey or failed an attention check (see Supplementary material), leaving 279. Participants answered all scales and tasks in a mean completion time of 9.8 min (*SD* = 6.6).

#### 3.2.2. Materials

The regressions reported below control for the variables collected in Study 1. In the reduced sample, 121 identified as male (43.4%), 152 as female (54.5%), and 6 as neither male nor female (2.2%). Mean age was 33.6 (*SD* = 12.7). 171 (61.3%) did not identify with a religious or spiritual group, whereas 108 (38.7%) did. Median education level was 4 out of 7 (college with associate’s degree) and median income level was 6 out of 13 (US$50,000–59,000).

We administered the 10-item Dual Foundations Scale, as refined by Study 1. Again, each participant completed the DFS twice: once about America and once about their family. We randomized the order of these versions. DFS items were presented in random order, using the same response technique as for Study 1. Mean responses for the DFS were: 25.44 (*SD* = 17.53) for Nation Inequality, 51.53 for Nation Social Control (*SD* = 14.26), 26.81 (*SD* = 14.65) for Family Inequality, and 45.02 (*SD* = 14.98) for Family Social Control. We report reliability statistics in the results.

We deployed the BEAST, a measure of reliance on social information in which participants guess the number of animals in three pictures (Molleman et al., 2019). Each participant is then shown another person’s estimate and the BEAST score represents how much they adjust their guess to match this social information. All BEAST scores vary between 0 and 100. If a participant did not move from their original estimate, their BEAST score was 0; if they moved their estimate to match the social information their score was 100. The mean was 29.77 (*SD* = 22.02). Details about the calculation of the BEAST score are provided in Supplementary material. We had also aimed to introduce a Public Goods Game as another incentivized measure of cooperation. Unfortunately, due to a procedural error, this did not collect usable data.

#### 3.2.3. Data analysis

We used the same statistical analysis techniques and R packages as for Study 1. Analysis code and de-identified data are also available (doi: 10.17605/OSF.IO/SYB9H).

#### 3.2.4. Ethics statement

Approval was granted by the University of Auckland Human Participants Ethics Committee on 04/03/2021 (ref. 024358).

### 3.3. Study 2 results

#### 3.3.1. Does the DFS produce two clear factors?

We first tested our pre-registered prediction that the second wave of DFS responses should support the dual foundations framework’s two dimensions. McDonald’s omegas were good: *ω* = 0.80 for Nation Inequality, *ω* = 0.77 for Nation Social Control, *ω* = 0.73 for Family Inequality, and *ω* = 0.80 for Family Social Control. In CFA, all items loaded positively and significantly onto their anticipated dimensions. Table 5 shows that in this new sample the two dimensions demonstrate good fit, and that responses from both studies can be combined and the two-dimension structure remains well-fitting. The Family wave 2 model shows CFI = 1 and RMSEA = 0; this model has an adequate number of degrees of freedom (34), indicating it is not miss-specified and simply fits well.

**Table 5:** Fit indices for models using the second wave of DFS data and both waves of DFS data.

Model	CFI	RMSEA	SRMR
DFS Nation wave 2: Inequality and social control dimensions	0.967	0.048	0.072
DFS Nation wave 2: Single dimension	0.530	0.181	0.174
DFS Nation wave 2: Pro-trait and con-trait dimensions	0.528	0.184	0.174
DFS Nation waves 1 and 2: Inequality and social control	0.963	0.046	0.077
DFS Family wave 2: Inequality and social control dimensions	1.000	0.000	0.056
DFS Family wave 2: Single dimension	0.754	0.117	0.141
DFS Family wave 2: Pro-trait and con-trait dimensions	0.754	0.119	0.140
DFS Family waves 1 and 2: Inequality and social control	0.967	0.039	0.078

CFI is the Comparative Fit Index, RMSEA is the Root Mean Square Error of Approximation, and SRMR is the Standardized Root Mean Residual.

#### 3.3.2. Does the DFS demonstrate measurement invariance between groups?

Table 6 supports our pre-registered predictions for measurement invariance. Model fit remains acceptable and the size of the difference in fit estimates between adjacent models with different invariance constraints is generally 0.01 or less. Again, the DFS Family models with CFI = 1 and RMSEA = 0 all show appropriate numbers of degrees of freedom (all between 68 and 84), indicating not miss-specification but good fit.

**Table 6:** Fit indices for DFS wave 2 models, with equality constraints for testing configural, metric, and scalar invariance.

Model	Invariance type	CFI	RMSEA	SRMR
Model 1 (*N* = 273)				
DFS Nation split by sex	Configural	0.993	0.024	0.077
DFS Nation split by sex	Metric	0.992	0.023	0.081
DFS Nation split by sex	Scalar	0.997	0.013	0.082
Model 2 (*N* = 279)				
DFS Nation split by age	Configural	0.972	0.046	0.082
DFS Nation split by age	Metric	0.971	0.045	0.086
DFS Nation split by age	Scalar	0.976	0.039	0.087
Model 3 (*N* = 279)				
DFS Nation split by ethnicity	Configural	0.982	0.038	0.078
DFS Nation split by ethnicity	Metric	0.968	0.047	0.088
DFS Nation split by ethnicity	Scalar	0.970	0.044	0.090
Model 4 (*N* = 273)				
DFS Family split by sex	Configural	1.000	0.000	0.065
DFS Family split by sex	Metric	1.000	0.000	0.071
DFS Family split by sex	Scalar	1.000	0.000	0.073
Model 5 (*N* = 279)				
DFS Family split by age	Configural	1.000	0.000	0.063
DFS Family split by age	Metric	1.000	0.000	0.066
DFS Family split by age	Scalar	1.000	0.000	0.068
Model 6 (*N* = 279)				
DFS Family split by ethnicity	Configural	1.000	0.000	0.064
DFS Family split by ethnicity	Metric	1.000	0.000	0.071
DFS Family split by ethnicity	Scalar	1.000	0.000	0.075

CFI is the Comparative Fit Index, RMSEA is the Root Mean Square Error of Approximation, and SRMR is the Standardized Root Mean Residual.

#### 3.3.3. Does the DFS demonstrate measurement invariance across time?

Table 7 shows that, as predicted, both DFS Nation and Family are invariant between waves 1 and 2: differences between adjacent models are all less than 0.01. This indicates that the DFS measures the two dimensions, in the same way, across the two waves.

**Table 7:** Fit indices for the DFS across waves, with equality constraints for testing configural, metric, and scalar invariance.

Model	Invariance type	CFI	RMSEA	SRMR
Nation (*N* = 279)				
DFS Nation split by wave	Configural	0.992	0.022	0.059
DFS Nation split by wave	Metric	0.987	0.028	0.063
DFS Nation split by wave	Scalar	0.980	0.033	0.066
Family (*N* = 279)				
DFS Family split by wave	Configural	1.000	0.000	0.052
DFS Family split by wave	Metric	1.000	0.000	0.055
DFS Family split by wave	Scalar	1.000	0.000	0.058

CFI is the Comparative Fit Index, RMSEA is the Root Mean Square Error of Approximation, and SRMR is the Standardized Root Mean Residual.

#### 3.3.4. Are participants’ DFS scores stable over time?

To investigate the between-wave stability of the two dimensions, we examine the correlations of the dimensions across the waves in the scalar invariance models. We find strong correlations between Inequality and Social Control scores across the two waves: the standardized correlation for Nation Inequality was 0.831 (95% CI [0.729, 0.933], *z* = 15.997, *p* < 0.001), for Nation Social Control 0.659 (95% CI [0.573, 0.745], *z* = 15.015, *p* < 0.001), for Family Inequality 0.674 (95% CI [0.550, 0.897], *z* = 10.634, *p* < 0.001), and for Family Social Control 0.696 (95% CI [0.618, 0.774], *z* = 17.461, *p* < 0.001). The 95% confidence intervals for each DFS dimension thus exclude all coefficients below 0.5. We note that this is an improved analysis of between-wave stability than the separate regression analysis we had pre-registered. It shows that participants held relatively stable opinions about the two DFS dimensions across the 7-month interval, indicating that they measure relatively stable constructs.

#### 3.3.5. Does the DFS show convergent and discriminant validity in relation to a new incentivized measure of conformity?

We analyzed BEAST scores in two ways. First, as the predictor variable in structural equation models, predicting our measures of social control values: DFS Nation Social Control, DFS Family Social Control, RWA, and Strict Father. Second, as the outcome variable, predicted by our political values measures in a multilevel Bayesian modeling framework. Details and results for both kinds of analysis are presented in Supplementary material. Neither style of analysis provided support for our predictions. In the structural equation models, the results with the DFS were the same as with RWA and Strict Father: the BEAST scores’ effects were small and non-significant. In the Bayesian models, none of the political values measures reliably predicted BEAST scores. In fact, aside from being negatively predicted by the round of the task, BEAST scores were not predicted by any variables included in the models. Some (not mutually exclusive) possibilities exist for why we did not replicate previous findings of a relationship between BEAST scores and RWA. Behavioral measures of politics-related constructs in general show smaller effect sizes in predicting political preferences than subjective self-evaluation measures, perhaps due to it being easier for individuals to express their preferences in semantic statements compared with evaluative tasks (Federico, 2022). Furthermore, our version of the BEAST may have exacerbated effect size issues. As described in Supplementary material, we adopted a different means of selecting the social information given to participants than prior work (Claessens et al., 2021), which somewhat reduced the gap between their initial estimate and the social information and thus provided less incentive for participants to update their scores. In addition, we sampled across three rather than the five original rounds of the BEAST, and our sample size was restricted by the limited number of individuals we were able to recontact in Study 2. The BEAST analyses may therefore have had problems detecting a small effect size.

## 4. Discussion

Our validation of the Dual Foundations Scale supports the dual foundations framework’s attempt to ground the study of political ideology in the trade-offs of group living. Based on evolutionary reasoning (Claessens et al., 2020), we created a scale that aims to reflect these two ubiquitous sources of political contestation. This scale shows that our participants do hold coherent, independent, and persistent values about the extent of inequality and social control. We thus find support for the hypothesis that two trade-offs, inherent to the evolution of human social life, are linked two dimensions of political values that we can measure with the Dual Foundations Scale.

We show that the DFS fares well in tests of internal reliability. The DFS’s items support the dual foundations framework’s predicted factor structure, they demonstrate measurement invariance between groups and across time, and they show within-individual stability across a seven-month period. Although our decision to recontact participants across the two studies means that we leave it to future work to demonstrate the generalizability of the DFS’s factor structure to other sample populations, recontacting the same participants also enabled us to demonstrate the DFS’s impressive stability across a politically tumultuous period of time, which involved a US Presidential election and change of premiership, as well as a global pandemic. Supporting the dual foundations framework’s prediction that ideological trade-offs exist in groups both big and small, the DFS performed as well in the “family politics” context as it did in the context of national politics. Moreover, the significant relationships between individuals’ responses across the family- and nation-levels support previous research indicating that people hold consistent preferences across both contexts (Feinberg et al., 2020). The DFS thus provides empirical support for the theoretical underpinnings of the dual foundations framework.

We also supported various hypotheses testing the DFS’s external validity in relation to existing scales of political ideology. This provides evidence, in the form of convergent and discriminant validity, that the ideological dimensions outlined by the dual foundations framework are those studied by past research. DFS Inequality seems to measure a similar construct to SDO and Nurturant Parent, which is clearly different from RWA and Strict Father. DFS Social Control seems to measure a similar construct to RWA and Strict Father, clearly distinct from SDO and Nurturant Parent. Furthermore, both DFS dimensions independently predict a general right-wing orientation. The results thus validate DFS Inequality and Social Control as accurate measures of two existing dimensions of ideological values, which independently contribute to the prevalent left-versus-right distinction in modern Western politics. Moreover, these findings support the dual foundations’ interpretation of SDO and Nurturant Parent scales as fundamentally capturing support for (or opposition to) inequality and RWA and Strict Father beliefs as fundamentally capturing support for social control.

A caveat of our findings is in the relationships between the politics scales and abstract incentivized tasks. The independent relationships demonstrated between ideological dimensions and Dictator Game Keepings and Rule Following do indicate that preferences in incentivized behavioral tasks are related to stated political values. However, we also report some unexpected results, such as the positive effects of Dictator Game Keepings on Strict Father and DFS Social Control Family. This may point toward more nuanced understandings of what participants perceive to be the social norm in these games and how this relates to their preferences regarding social control as well as fairness. Another surprising result was the lack of significant relationships between the BEAST and RWA, Strict Father, and DFS Social Control. Again, this evidence points to relationships between the behavioral tasks and politics scales being complex in general, rather than a problem with the DFS itself. Although we chose tasks that past research shows are related to SDO and RWA (Claessens et al., 2021; Fischer et al., 2021), this being a new area of research, the tasks have not yet been validated through years of consistent findings. For example, while some work finds rule following relates to RWA and we find some supportive evidence, Claessens et al. (2021) do not find a relationship. We thus emphasize that our results with the behavioral tasks should be taken as a steppingstone toward better understanding of the relation of political values to behavior in incentivized tasks, as this is a field which is only beginning to uncover the connections between stated values and behavioral preferences. Moreover, our finding that the DFS shows similar patterns of relationships to behavioral tasks as the other politics scales supports our hypothesis that they capture the same underlying preferences.

Our findings question the straightforward relationship between Schwartz values and the two dimensions of political values. First, in contrast to Schwartz et al. (2012, p. 675), some PVQ values on orthogonal and opposite sides of the value circle correlated positively. Second, of those values that did relate to other values as anticipated, some predicted both dimensions of political values, making them less than ideal measures of the two dimensions. This was as true with SDO-RWA and Nurturant Parent^R^-Strict Father as it was with the DFS. A possible explanation is that we used Schwartz’s refined values, in contrast to previous politics research that uses older questionnaires which divide the value continuum into fewer dimensions (Schwartz et al., 2014; Caprara et al., 2017). Researchers may therefore want to continue to use the broader value dimensions. This may, however, hide the complexities we found by analyzing Schwartz’s refined values. We therefore recommend further research on the relation between refined Schwartz values and political ideology’s two dimensions. This research may uncover more nuanced and perhaps surprising relationships between ideology and personal values.

The primary implication of our findings is validation of the dual foundations approach to the two dimensions of ideology. We have demonstrated, in our US sample, the DFS’s ability to measure the two dimensions of political ideology in a manner predicted by recent theoretical arguments that two broad dimensions of ideology reflect fundamental trade-offs in human group-living. A key advantage of the DFS is therefore its ability to remove the current gap between theory and operationalization, since the two dimensions are most often measured by scales created for other purposes. While SDO measures attitudes to group dominance and RWA measures *right-wing* authoritarianism, the DFS is an explicitly two-dimensional scale that measures specific political values regarding inequality and social control. The DFS’s clarity in operationalizing the two dimensions derives from posing the trade-offs of social life identified by the dual foundations framework. Our work here supports this operationalization as a useful measure of the two dimensions of political ideology.

Another key advantage of the DFS—the ability to substitute different social groups into the same scale—opens two key avenues for future research. First, the DFS allows psychologists to study the two dimensions of political values across different levels of social organization within one society. The DFS’s short length means that several versions of the scale can be administered in a relatively short space of time. This allows for more detailed analysis of the consistency and variation of individuals’ political attitudes in relation to various social groups, thus providing insight into whether political opinions are more flexible to particular contextual features (e.g., Nettle and Saxe, 2020) or more context-insensitive outcomes of prior psychological dispositions (e.g., Duckitt and Sibley, 2009). Second, the DFS constitutes a candidate for expanding political psychology research beyond samples from industrialized contexts and centralized nation-states. The flexibility of the DFS, where any social group can be swapped in and its items should retain meaning and relevance, makes it a promising tool for exploring cross-cultural variation in the structure of political values.

In conclusion, we have shown that, within an American online sample, the Dual Foundations Scale is an accurate and reliable measure of the two dimensions of ideological values. Our research supports the dual foundations framework’s argument that the two dimensions of politics are rooted in trade-offs inherent to human social life. The DFS’s theoretical rationale, together with its properties, including succinctness, avoidance of “hot-button” issues, and flexibility to different social contexts, make it a good candidate for further work in political psychology and extension of this work to different societies.

## Data availability statement

The datasets presented in this study can be found in online repositories. The names of the repository/repositories and accession number(s) can be found at: doi: 10.17605/OSF.IO/SYB9H.

## Ethics statement

The studies involving human participants were reviewed and approved by the University of Auckland Human Participants Ethics Committee. The participants provided online informed consent.

## Author contributions

GL: substantial contributions to study design, data collection, statistical analysis, and manuscript drafting and editing. QA: substantial contribution to study design, statistical analysis, and manuscript editing. AC: contributions to study design and manuscript writing. All authors contributed to the article and approved the submitted version.

## Funding

This project was funded by the RSNZ Marsden Fund grant awarded to QA and AC (code: UOA1711).

## Conflict of interest

The authors declare that the research was conducted in the absence of any commercial or financial relationships that could be construed as a potential conflict of interest.

## Publisher’s note

All claims expressed in this article are solely those of the authors and do not necessarily represent those of their affiliated organizations, or those of the publisher, the editors and the reviewers. Any product that may be evaluated in this article, or claim that may be made by its manufacturer, is not guaranteed or endorsed by the publisher.

## Footnotes

^1^ https://www.prolific.co/

^2^ https://www.qualtrics.com/au/
