# Supplementary material for: Validating the dual evolutionary foundations of political values in a US sample
Source: Front Psychol. 2023 Jun 23;14:1189771. doi: 10.3389/fpsyg.2023.1189771 (PMC10326618; doi:10.3389/fpsyg.2023.1189771)
Supplement: Supplementary file 1 [file Data_Sheet_1.pdf]

Supplementary Material

1 Attention checks

We included two attention check questions in Study 1 and one in Study 2. All attention checks asked participants to put the slider button in the middle of the scale. We judged participants were not paying sufficient attention if they failed to place the slider near the centre, defined as values above 34 and below 66 on the 100-point scale. We excluded any participant that gave a value outside that range for either attention check.

2 Ideology scale items

Supplementary Table 1

Items for Social Dominance Orientation and Right Wing Authoritarianism

Dimension	Wording	Valence
SDO	Some groups of people must be kept in their place.	Pro-trait
	It's probably a good thing that certain groups are at the top and other groups are at the bottom.	Pro-trait
	An ideal society requires some groups to be on top and others to be on the bottom.	Pro-trait
	Some groups of people are simply inferior to other groups.	Pro-trait
	Groups at the bottom are just as deserving as groups at the top.	Con-trait
	No one group should dominate in society.	Con-trait
	Groups at the bottom should not have to stay in their place.	Con-trait
	Group dominance is a poor principle.	Con-trait
	We should not push for group equality.	Pro-trait
	We shouldn't try to guarantee that every group has the same quality of life.	Pro-trait
	It is unjust to try to make groups equal.	Pro-trait
	Group equality should not be our primary goal.	Pro-trait
	We should work to give all groups an equal chance to succeed.	Con-trait
	We should do what we can to equalize conditions for different groups.	Con-trait
	No matter how much effort it takes, we ought to strive to ensure that all groups have the same chance in life.	Con-trait
	Group equality should be our ideal.	Con-trait
RWA	It's great that many young people today are prepared to defy authority.	Con-trait
	What our country needs most is discipline, with everyone following our leaders in unity.	Pro-trait
	Students at high schools and at university must be encouraged to challenge, criticize, and confront established authorities.	Con-trait
	Obedience and respect for authority are the most important virtues children should learn.	Pro-trait
	Our country will be great if we show respect for authority and obey our leaders.	Pro-trait
	People should be ready to protest against and challenge laws they don't agree with.	Con-trait
	Nobody should stick to the "straight and narrow." Instead people should break loose and try out lots of different ideas and experiences.	Con-trait
	The "old-fashioned ways" and "old-fashioned values" still show the best way to live.	Pro-trait
	God's laws about abortion, pornography, and marriage must be strictly followed before it is too late.	Pro-trait
	There is absolutely nothing wrong with nudist camps.	Con-trait
	This country will flourish if young people stop experimenting with drugs, alcohol, and sex, and pay more attention to family values.	Pro-trait
	There is nothing wrong with premarital sexual intercourse.	Con-trait
	Strong, tough government will harm not help our country.	Con-trait
	Being kind to loafers or criminals will only encourage them to take advantage of your weakness, so it's best to use a firm, tough hand when dealing with them.	Pro-trait
	Our society does NOT need tougher government and stricter laws.	Con-trait
	The facts on crime and the recent public disorders show we have to crack down harder on troublemakers, if we are going preserve law and order.	Pro-trait
	Our prisons are a shocking disgrace. Criminals are unfortunate people who deserve much better care, instead of so much punishment.	Con-trait
	The way things are going in this country, it's going to take a lot of "strong medicine" to straighten out the troublemakers, criminals, and perverts.	Pro-trait

Note: SDO items from Ho et al. (2015), RWA items from Duckitt et al. (2010).

Supplementary Table 2

Items for Nurturant Parent (reversed) and Strict Father

Dimension	Wording	Valence
NP (r)	Children must learn to see the world through other people's eyes.	Con-trait
	Parenting means nurturing the child's true nature.	Con-trait
	Knowing how to care for others is not a central thing for a child to learn.	Pro-trait
	Children will grow up to be happy adults if parents encourage them to follow their curiosity.	Con-trait
	In order to truly nurture children one needs to be empathic.	Con-trait
	Learning to understand others and accepting them for who they are is not important for children to learn.	Pro-trait
	Children shouldn't feel obligated to care about the well-being of people they do not know.	Pro-trait
	Siblings should receive parental support in accordance to their individual needs.	Con-trait
	I'd rather see my child play cooperatively than play competitively.	Con-trait
	Tending to the needs of others is not a sign of responsibility in children.	Pro-trait
	Children should learn to understand others needs and attend to them.	Con-trait
	Parents should empower children as much as possible so that they may follow their dreams.	Con-trait
	It's not important for parents to explain to their children why they set certain rules and limits.	Pro-trait
	It's not critical for children to learn to take the perspective of others into account.	Pro-trait
SF	Children must be disciplined through strict rules at home.	Con-trait
	Bad behavior in children must be punished sufficiently.	Con-trait
	I will not have my child talk back to me.	Con-trait
	Sometimes it's okay to let bad behavior in children go unpunished.	Pro-trait
	Children need to be disciplined in order to build character.	Con-trait
	Obedience must be instilled in children	Con-trait
	When grownups talk children ought to be quiet.	Con-trait
	It's fine for children to have secrets and hide things from their parents	Pro-trait
	"Tough love" is required to raise a child right.	Con-trait
	Children must always be on time.	Con-trait
	When in doubt, parents should err on the side of lenience rather than strictness.	Pro-trait
	Parents shouldn't handicap their children by making their lives too easy.	Con-trait
	While other people must not be one's concern, within a family, everyone should look after each other.	Con-trait
	Children must be taught that people get what they deserve.	Con-trait
	At times it's okay for children to disobey their parents.	Pro-trait

Note: NP and SF items from Feinberg et al. (2020).

Supplementary Table 3

Selected items from Schwartz's refined Portrait Values Questionnaire

Dimension	Name	Wording	Valence
Inequality	Benevolence-Caring (r)	It’s very important to this person to help the people dear to them.	Con-trait
		Caring for the well-being of people they are close to is important to this person.	Con-trait
		It is important to this person to be loyal to those who are close to them.	Con-trait
	Benevolence-Dependability (r)	This person goes out of their way to be a dependable and trustworthy friend.	Con-trait
		This person wants those they spend time with to be able to rely on them completely.	Con-trait
	Universalism-Concern (r)	Protecting society’s weak and vulnerable members is important to this person.	Con-trait
		This person thinks it is important that every person in the world have equal opportunities in life.	Con-trait
		This person wants everyone to be treated justly, even people they don’t know.	Con-trait
	Achievement	This person thinks it is important to be ambitious.	Pro-trait
		Being very successful is important to this person.	Pro-trait
		This person wants people to admire their achievements.	Pro-trait
Social Control	Tradition	It is important to this person to maintain traditional values or beliefs.	Pro-trait
		Following their family’s customs or the customs of a religion is important to this person.	Pro-trait
		This person strongly values the traditional practices of their culture.	Pro-trait
	Conformity-Rules	It is important to this person to follow rules even when no one is watching.	Pro-trait
		Obeying all the laws is important to this person.	Pro-trait
	Self-Direction-Thought (r)	Being creative is important to this person.	Con-trait
		It is important to this person to form their own opinions and have original ideas.	Con-trait
		Learning things for themselves and improving their abilities is important to this person.	Con-trait
	Self-Direction-Action (r)	It is important to this person to make their own decisions about their life.	Con-trait
		Doing everything independently is important to this person	Con-trait
		Freedom to choose what they do is important to this person.	Con-trait

Note: PVQ items from Schwartz et al. (2012).

3 Calculation of BEAST scores

The BEAST (Berlin Estimate AdjuStment Task) works by giving each participant two attempts to guess how many animals are displayed on their computer screen. For their first guess, the participant was given 6 seconds to look at the picture and 30 seconds to enter a number. They were then told a guess made by somebody who had previously participated in the task and were given a window of 45 seconds to enter another estimate. The social information we provided to them was dependent on their initial estimate. We did this by using a list of guesses made by a previous set of BEAST participants and finding the one which was closest to the participant's first estimate. If this estimate was below the true number of animals, we counted seven previous guesses higher on this list and that response was given to the participant as the social information. If the participant's first estimate was above the true number of animals, we counted down seven previous guesses on the list and gave that as the social information. The same procedure was used for the other two rounds, though using different lists of previous participants' guesses. This method of determining social information differs from that used by Molleman et al. (2019) because we aimed to simplify the task for use in later in-person fieldwork. Before conducting the study, we calculated differences between our strategy for selecting the social information and the equation used by Molleman et al. Our method selects social information which is similar to that of Molleman et al.'s equation, although a little less extreme. It selects social information which is most similar to Molleman et al.'s equation at the centre of the likely range of participant guesses and becomes increasingly more conservative than Molleman's equation at extremely low or high guesses relative to the true number of animals. The lists of previous participants' guesses which were used as the social information were data collected for a previous study in the USA (Claessens *et al.*, 2021). To calculate the participant's BEAST score for each round, we used this formula to arrive at a score between 0 and 100:

BEAST score = ((Second estimate – First estimate) / (Social information – First estimate)) * 100

We take the mean of the three rounds as a single BEAST score. This is fewer than the five rounds used by Molleman et al. Following Molleman et al., we remove rounds in which a participant's second estimate moved away from the social information, moved further than the social information, or in which no estimate was given. Thus 39 participants' BEAST scores represent two rather than three rounds, 9 participants' scores represent one round, and 2 participants were dropped in these analyses for lack of a BEAST score.

4 DFS item correlations

Supplementary Table 4

Items for DFS Nation and DFS Family

Dimension	Item	Wording	Valence	Inter-item correlation 1	Inter-item correlation 2
Nation Inequality	1	When some Americans get a lot of resources, they use most of their resources for their own personal benefit.	Pro-trait	0.329	
	**2**	**When some Americans get a lot of resources, they do not share any of their resources with other Americans.**	**Pro-trait**	**0.355**	**0.468**
	3	When some Americans get some resources, they use their resources for the good of other Americans.	Con-trait	0.300	
	**4**	**When some Americans get a lot of resources, they share all of these resources with other Americans.**	**Con-trait**	**0.323**	**0.428**
	5	Some Americans try to get more resources than other Americans.	Pro-trait	0.260	
	**6**	**Some Americans do not get the resources that other Americans have, which means that some Americans have less than others.**	**Pro-trait**	**0.365**	**0.503**
	7	Some Americans try to make sure that no Americans get more resources than other Americans.	Con-trait	0.226	
	**8**	**Some Americans try to make sure that no Americans get fewer resources than other Americans.**	**Con-trait**	**0.323**	**0.458**
	9	A small group of Americans can make other Americans do things they don't want to do.	Pro-trait	0.203	
	10	A small group of Americans can do things that do not benefit the rest of America and which other Americans do not want them to do.	Pro-trait	0.257	
	11	Some Americans try to take away the ability of a small group of Americans to make other Americans do things they don't want to do.	Con-trait	-0.013	
	12	Some Americans try to stop a small number of Americans getting too much control over other Americans.	Con-trait	0.279	
Nation Social Control	**13**	**All Americans have to follow all of the USA's rules all of the time.**	**Pro-trait**	**0.331**	**0.401**
	14	Some Americans try to stop other Americans from changing lots of the USA's rules.	Pro-trait	0.191	
	**15**	**An American does not follow some of the USA's rules because they do not agree with them.**	**Con-trait**	**0.303**	**0.409**
	16	All Americans should question the USA's rules all of the time.	Con-trait	0.316	
	17	The USA punishes an American very harshly, because they have broken a US rule which you think is important.	Pro-trait	0.183	
	**18**	**The USA punishes an American very harshly, because they have repeatedly broken the USA's rules.**	**Pro-trait**	**0.252**	**0.270**
	**19**	**The USA does not punish an American after they have broken a US rule which you don't think is important.**	**Con-trait**	**0.227**	**0.244**
	20	The USA does not punish an American, because they broke a US rule for the first time.	Con-trait	0.180	
	**21**	**Some Americans don't agree with a decision made by the USA's leaders, but they have to follow the leaders' decision anyway.**	**Pro-trait**	**0.232**	**0.296**
	22	Some Americans don't want other Americans criticizing the USA's leaders.	Pro-trait	0.246	
	**23**	**An American does not follow the decision of the USA's leaders, because they don't agree with the leaders' decision.**	**Con-trait**	**0.290**	**0.351**
	24	Some Americans think they should always question whether the USA's leaders are making the right decisions.	Con-trait	0.272	
Family Inequality	1	When some family members get a lot of resources, they use most of their resources for their own personal benefit.	Pro-trait	0.262	
	**2**	**When some family members get a lot of resources, they do not share any of their resources with other family members.**	**Pro-trait**	**0.324**	**0.447**
	3	When some family members get some resources, they use their resources for the benefit of other family members.	Con-trait	0.305	
	**4**	**When some family members get a lot of resources, they share all of these resources with other family members.**	**Con-trait**	**0.320**	**0.420**
	5	Some members of your family try to get more resources than other family members.	Pro-trait	0.246	
	**6**	**Some of your family members do not get the resources that other family members have, which means that some family members have less than others.**	**Pro-trait**	**0.301**	**0.413**
	7	Some members of your family try to make sure that no family members get more resources than other family members.	Con-trait	0.223	
	**8**	**Some members of your family try to make sure that no family members get fewer resources than other family members.**	**Con-trait**	**0.316**	**0.428**
	9	A small group of family members can make other members of your family do things they don't want to do.	Pro-trait	0.176	
	10	A small group of family members can do things that do not benefit the rest of your family and which other members of your family do not want them to do.	Pro-trait	0.219	
	11	Some members of your family try to take away the ability of a small group of family members to make other members of your family do things they don't want to do.	Con-trait	-0.015	
	12	Some members of your family try to stop a small number of family members getting too much control over other members of your family.	Con-trait	0.212	
Family Social Control	**13**	**All members of your family have to follow all of the family's rules all of the time.**	**Pro-trait**	**0.336**	**0.400**
	14	Some members of your family try to stop other family members from changing lots of the family's rules.	Pro-trait	0.221	
	**15**	**A family member does not follow some of your family's rules because they do not agree with them.**	**Con-trait**	**0.315**	**0.401**
	16	All members of your family should question the family's rules all of the time.	Con-trait	0.311	
	17	Your family punishes a family member very harshly, because they have broken a family rule which you think is important.	Pro-trait	0.256	
	**18**	**Your family punishes a family member very harshly, because they have repeatedly broken your family's rules.**	**Pro-trait**	**0.267**	**0.311**
	**19**	**Your family does not punish a family member after they have broken a family rule which you don't think is important.**	**Con-trait**	**0.250**	**0.281**
	20	Your family does not punish a family member, because they broke a family rule for the first time.	Con-trait	0.221	
	**21**	**Some members of your family don't agree with a decision made by the family's leaders, but they have to follow the leaders' decision anyway.**	**Pro-trait**	**0.328**	**0.377**
	22	Some of your family members don't want other family members criticizing the family's leaders.	Pro-trait	0.278	
	**23**	**A family member does not follow the decision of your family's leaders, because they don't agree with the leaders' decision.**	**Con-trait**	**0.311**	**0.377**
	24	Some members of your family think they should always question whether your family's leaders are making the right decisions.	Con-trait	0.276	

Note: Bold indicates the item was retained.

Following the item reduction process presented in Supplementary Table 4, Nation Inequality's mean inter-item correlation was 0.46 (SD=0.03), Nation Social Control's mean inter-item correlation was 0.33 (SD=0.07), Family Inequality's mean inter-item correlation was 0.43 (SD=0.01), and Family Social Control's mean inter-item correlation was 0.36 (SD=0.05).

5 Exploratory analyses of relationships between the two dimensions of ideology

Supplementary Figure 1 presents results of a non-preregistered analysis that investigates how strongly related each of the two dimensions of ideological values are to each other. These models all control for demographic covariates and religion. Model 1 predicts DFS Nation Social Control from DFS Nation Inequality, Model 2 predicts DFS Family Social Control from DFS Family Inequality, Model 3 predicts RWA from SDO, and Model 4 predicts Strict Father from Nurturant Parent (reversed).

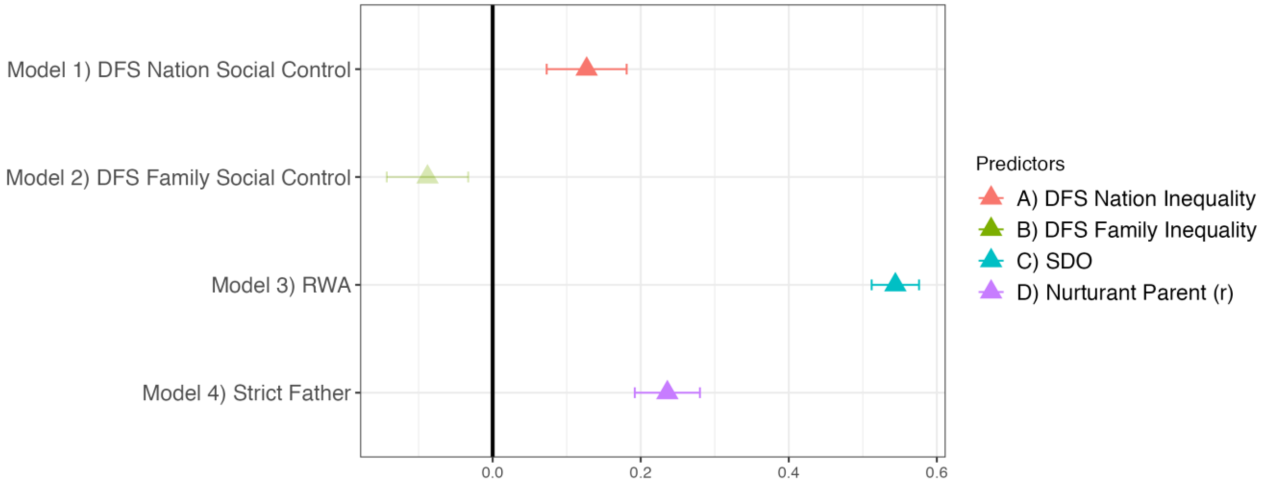

Supplementary Figure 1. Relationships between various measures of ideology's two dimensions. The outcome variable of each model is given on the y axis, with the predictors' effects plotted on the x axis. Translucent points indicate $p \geq 0.05$. Regression estimates are standardised betas with standard errors. All four models control for demographic covariates and religion.

6 Explanation of the exclusion of three Schwartz PVQ values

In the main text, we describe how, of the eight Schwartz PVQ values for which we made pre-registered predictions, three did not behave as anticipated. We therefore do not present analyses for these Schwartz values in the main text. Here, we will explain the process by which we decided to exclude these values from the main analysis.

If the 'Achievement' value represented a competitive motivation (indeed it is opposed to the Benevolence section on Schwartz's value circumplex), we would expect it to correlate negatively with Benevolence-Caring, Benevolence-Dependability, and Universalism-Concern. Similarly, we would expect Self-Direction-Thought and -Action to correlate negatively with the Tradition and

Conformity-Rules values, given their opposition to each other in Schwartz's value circumplex. Supplementary Figure 2 presents a correlation matrix using Spearman's rank. It displays the strengths of correlations between each of the eight PVQ values and shows that Achievement correlates positively with Benevolence-Caring, Benevolence-Dependability, and Universalism-Concern. Furthermore, we find no negative correlations between the Self-Direction values and Tradition and Conformity-Rules. Supplementary Figure 3 presents a results of an exploratory factor analysis (EFA; with oblimin rotation, using the *psych* package), showing how the same eight values load onto two factors. This plot does provide our expected two-dimensional structure with Tradition and Conformity-Rules clustering together, and Universalism-Concern, Benevolence-Dependability, and Benevolence-Caring clustering together. However, the EFA also shows that Self-Direction-Thought and -Action load positively to this latter dimension, as does Achievement. Because Achievement, Self-Direction-Thought, and Self-Direction-Action therefore did not behave the way we expected them to, the predictions we made about them in the pre-registration are uninterpretable. The pre-registered analyses are therefore presented below, rather than in the main text, and we do not hold them to be tests of the theory.

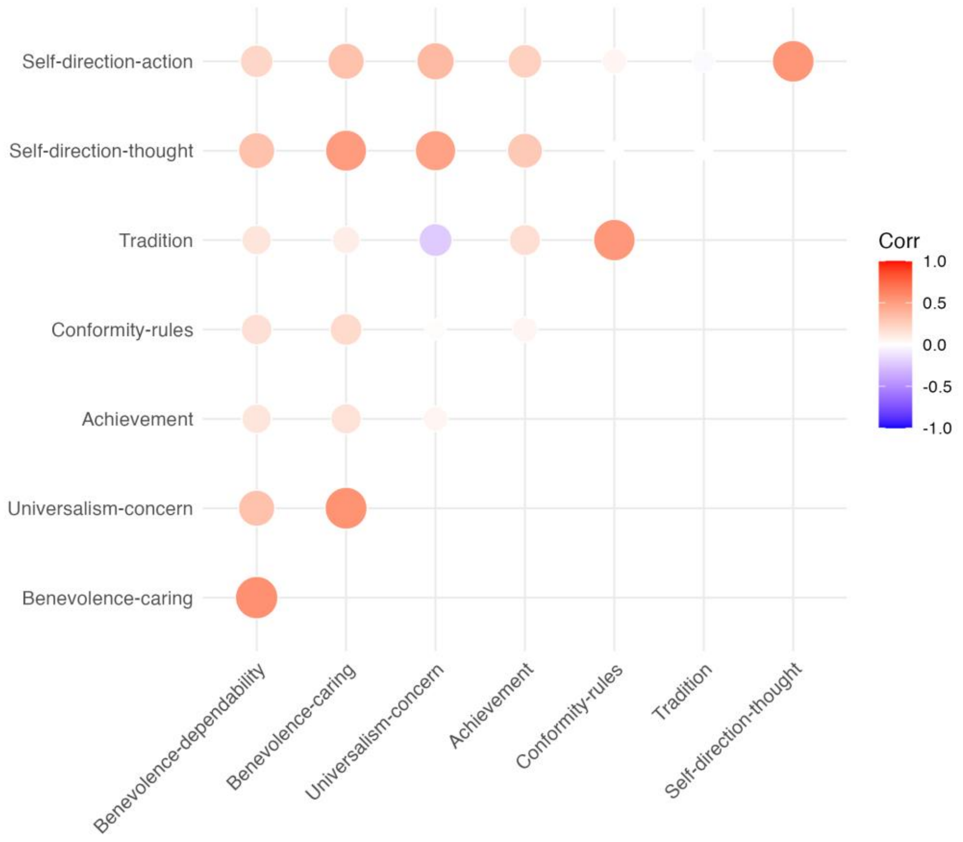

Supplementary Figure 2. Correlation matrix of eight Schwartz PVQ values. Red indicates a positive correlation, blue a negative correlation.

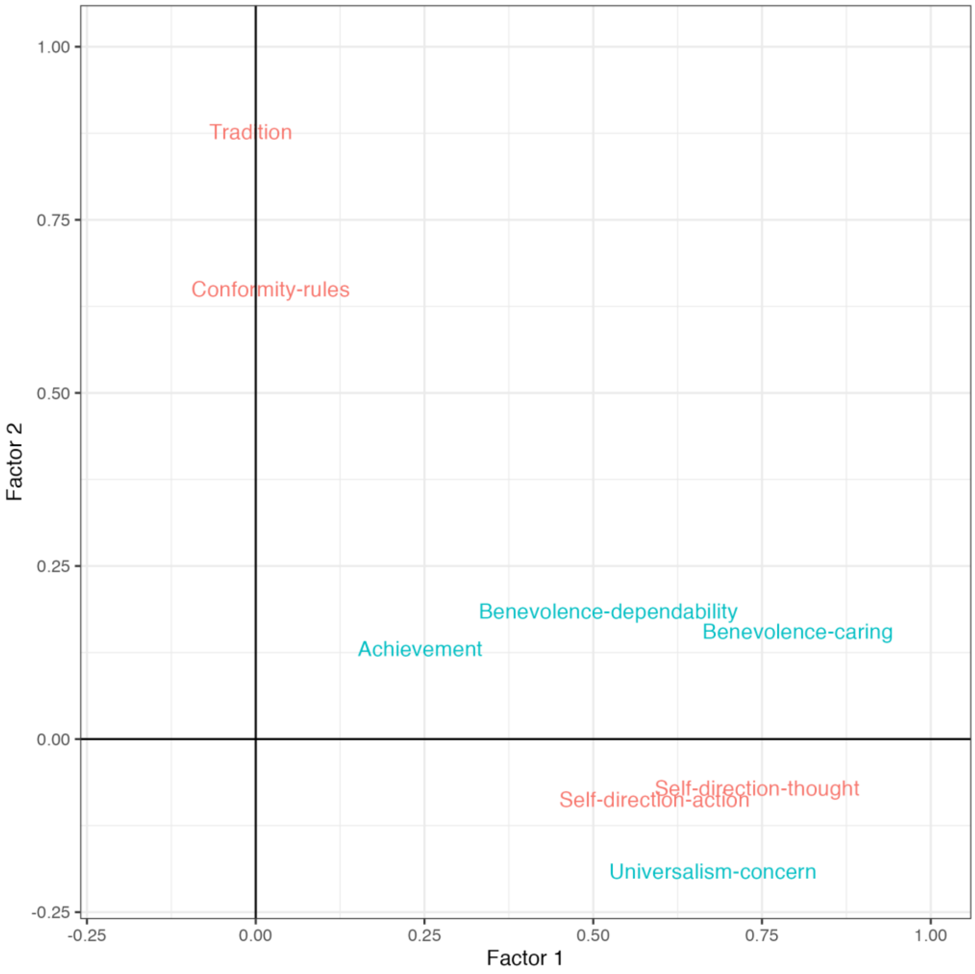

Supplementary Figure 3. EFA results for eight Schwartz PVQ values loading on two dimensions. Values we anticipated to load onto a dimension about inequality are coloured blue, and values we anticipated to load onto a dimension about social control are coloured red.

7 Pre-registered analyses predicting the DFS dimensions from Schwartz PVQ values

Supplementary Table 5 provides regression estimates for the effects of Schwartz's PVQ values on the DFS dimensions. We present these results in the form of a table to ease visualisation with the number of predictors and outcome variables. These models all control for demographic covariates and religion. We find that DFS Inequality (both Nation and Family) is predicted by Benevolence-Caring^R (S_BEC), Benevolence-Dependability^R (S_BED), and Universalism-Concern^R (S_BEC). We find that DFS Social Control (both Nation and Family) is predicted by Tradition (S_TR) and Conformity-Rules (S_COR). We also find some more unexpected results. Tradition positively predicts DFS Nation Inequality as well, but not Family Inequality. Universalism-Concern^R also sometimes quite strongly predicts DFS Social Control (both Nation and Family).

Supplementary Table 5

Regression estimates for the effects of Schwartz values on the DFS, controlling for demographic covariates and religion

Outcome	Predictor	Standardised beta	SE	95% CI	Z	p-value
**Model 1 (N=489)**						
Inequality_nat	S_BEC	0.372	0.043	0.288, 0.456	8.669	0.000
	S_TR	0.321	0.061	0.201, 0.44	5.265	0.000
	S_COR	0.057	0.054	-0.05, 0.164	1.046	0.295
**Model 2 (N=489)**						
Inequality_nat	S_BED	0.196	0.047	0.103, 0.288	4.148	0.000
	S_TR	0.308	0.064	0.183, 0.433	4.821	0.000
	S_COR	0.036	0.057	-0.075, 0.147	0.635	0.525
**Model 3 (N=489)**						
Inequality_nat	S_UNC	0.517	0.038	0.442, 0.592	13.531	0.000
	S_TR	0.168	0.059	0.051, 0.285	2.825	0.005
	S_COR	0.071	0.051	-0.029, 0.172	1.392	0.164
**Model 4 (N=489)**						
Social_control_nat	S_TR	0.401	0.054	0.295, 0.508	7.374	0.000
	S_BEC	-0.049	0.063	-0.173, 0.075	-0.779	0.436
	S_BED	-0.052	0.056	-0.161, 0.058	-0.927	0.354
	S_UNC	0.105	0.056	-0.006, 0.215	1.856	0.063
**Model 5 (N=489)**						
Social_control_nat	S_COR	0.523	0.038	0.448, 0.598	13.668	0.000
	S_BEC	-0.054	0.058	-0.168, 0.06	-0.923	0.356
	S_BED	-0.062	0.051	-0.163, 0.039	-1.209	0.227
	S_UNC	0.167	0.050	0.068, 0.266	3.320	0.001
**Model 6 (N=489)**						
Inequality_fam	S_BEC	0.436	0.044	0.349, 0.523	9.800	0.000
	S_TR	0.093	0.068	-0.039, 0.225	1.377	0.169
	S_COR	0.061	0.059	-0.054, 0.176	1.040	0.298
**Model 7 (N=489)**						
Inequality_fam	S_BED	0.177	0.052	0.076, 0.278	3.421	0.001
	S_TR	0.063	0.072	-0.077, 0.204	0.880	0.379
	S_COR	0.034	0.062	-0.087, 0.155	0.548	0.583
**Model 8 (N=489)**						
Inequality_fam	S_UNC	0.497	0.043	0.413, 0.582	11.581	0.000
	S_TR	-0.061	0.067	-0.193, 0.07	-0.913	0.361
	S_COR	0.070	0.057	-0.043, 0.182	1.212	0.225
**Model 9 (N=489)**						
Social_control_fam	S_TR	0.416	0.053	0.312, 0.52	7.861	0.000
	S_BEC	-0.064	0.062	-0.185, 0.057	-1.031	0.303
	S_BED	-0.073	0.054	-0.18, 0.033	-1.351	0.177
	S_UNC	0.175	0.055	0.068, 0.282	3.203	0.001
**Model 10 (N=489)**						
Social_control_fam	S_COR	0.336	0.045	0.248, 0.423	7.524	0.000
	S_BEC	-0.096	0.061	-0.216, 0.024	-1.566	0.117
	S_BED	-0.095	0.054	-0.201, 0.011	-1.750	0.080
	S_UNC	0.252	0.052	0.149, 0.355	4.811	0.000

8 Exploratory analyses predicting SDO-RWA and Nurturant Parent^R-Strict Father from the Schwartz PVQ values

Supplementary Table 6 provides regression estimates for the effects of the Schwartz PVQ values on the SDO-RWA and Nurturant Parent^R-Strict Father. These models all control for demographic covariates and religion. We find that both SDO and Nurturant Parent^R are predicted by Benevolence-Caring^R, Benevolence-Dependability^R, and Universalism-Concern^R. We find that both RWA and Strict Father are predicted by Tradition and Conformity-Rules. We also find some unexpected results: Universalism-Concern^R positively predicts both RWA and Strict Father, while Tradition positively predicts both SDO and Nurturant Parent^R.

Supplementary Table 6

Regression estimates for the effects of Schwartz Values on SDO/RWA and NP/SF, controlling for demographic covariates and religion

Outcome	Predictor	Standardised beta	SE	95% CI	Z	p-value
**Model 1 (N=489)**						
SDO	S_BEC	0.325	0.037	0.253, 0.397	8.807	0.000
	S_TR	0.403	0.050	0.304, 0.501	7.994	0.000
	S_COR	0.037	0.046	-0.053, 0.128	0.803	0.422
**Model 2 (N=489)**						
SDO	S_BED	0.240	0.039	0.164, 0.317	6.176	0.000
	S_TR	0.404	0.052	0.302, 0.506	7.768	0.000
	S_COR	0.022	0.048	-0.071, 0.115	0.458	0.647
**Model 3 (N=489)**						
SDO	S_UNC	0.627	0.025	0.579, 0.675	25.605	0.000
	S_TR	0.230	0.043	0.147, 0.314	5.405	0.000
	S_COR	0.069	0.037	-0.004, 0.141	1.850	0.064
**Model 4 (N=489)**						
RWA	S_TR	0.631	0.032	0.568, 0.694	19.633	0.000
	S_BEC	-0.025	0.041	-0.106, 0.056	-0.609	0.542
	S_BED	-0.016	0.036	-0.088, 0.055	-0.453	0.651
	S_UNC	0.241	0.036	0.171, 0.312	6.682	0.000
**Model 5 (N=489)**						
RWA	S_COR	0.456	0.030	0.397, 0.515	15.160	0.000
	S_BEC	-0.080	0.044	-0.165, 0.006	-1.817	0.069
	S_BED	-0.053	0.039	-0.129, 0.023	-1.363	0.173
	S_UNC	0.364	0.036	0.293, 0.435	10.032	0.000
**Model 6 (N=489)**						
Nurturant_parent	S_BEC	0.555	0.031	0.494, 0.615	17.962	0.000
	S_TR	0.462	0.048	0.368, 0.556	9.617	0.000
	S_COR	-0.079	0.044	-0.167, 0.008	-1.786	0.074
**Model 7 (N=489)**						
Nurturant_parent	S_BED	0.354	0.039	0.277, 0.431	9.061	0.000
	S_TR	0.455	0.054	0.35, 0.561	8.466	0.000
	S_COR	-0.110	0.050	-0.207, -0.013	-2.217	0.027
**Model 8 (N=489)**						
Nurturant_parent	S_UNC	0.590	0.030	0.531, 0.649	19.566	0.000
	S_TR	0.273	0.050	0.175, 0.371	5.475	0.000
	S_COR	-0.072	0.044	-0.157, 0.014	-1.645	0.100
**Model 9 (N=489)**						
Strict_father	S_TR	0.540	0.039	0.462, 0.617	13.688	0.000
	S_BEC	-0.189	0.048	-0.283, -0.096	-3.956	0.000
	S_BED	0.040	0.043	-0.044, 0.123	0.929	0.353
	S_UNC	0.240	0.042	0.157, 0.323	5.668	0.000
**Model 10 (N=489)**						
Strict_father	S_COR	0.420	0.034	0.353, 0.487	12.282	0.000
	S_BEC	-0.232	0.048	-0.326, -0.137	-4.814	0.000
	S_BED	0.010	0.043	-0.075, 0.095	0.234	0.815
	S_UNC	0.341	0.041	0.262, 0.421	8.382	0.000

9 Pre-registered analyses of the relationship between the DFS and omitted Schwartz values

Since the omitted Schwartz values did not measure what we expected them to, our pre-registered predictions about them are not interpretable. Nevertheless, Supplementary Tables 7 and 8 present the results of those pre-registered analyses. As might be expected from the findings above, these results are generally not in line with the pre-registration.

Supplementary Table 7

Regression estimates for the DFS predicting the omitted Schwartz Values, controlling for demographic covariates and religion

Outcome	Predictor	Standardised beta	SE	95% CI	Z	p-value
**Model 1 (N=489)**						
S_AC	Inequality_nat	0.000	0.050	-0.099, 0.099	0.000	1.000
	Social_control_nat	0.136	0.049	0.04, 0.232	2.787	0.005
**Model 2 (N=489)**						
S_SDA	Social_control_nat	0.001	0.051	-0.099, 0.101	0.021	0.983
	Inequality_nat	0.147	0.052	0.045, 0.248	2.838	0.005
**Model 3 (N=489)**						
S_SDT	Social_control_nat	0.011	0.050	-0.087, 0.11	0.224	0.822
	Inequality_nat	0.259	0.050	0.162, 0.357	5.191	0.000
**Model 4 (N=489)**						
S_AC	Inequality_fam	-0.054	0.050	-0.152, 0.045	-1.073	0.283
	Social_control_fam	0.101	0.048	0.008, 0.194	2.128	0.033
**Model 5 (N=489)**						
S_SDA	Social_control_fam	0.148	0.048	0.054, 0.242	3.074	0.002
	Inequality_fam	0.223	0.050	0.125, 0.321	4.461	0.000
**Model 6 (N=489)**						
S_SDT	Social_control_fam	0.098	0.048	0.005, 0.192	2.057	0.040
	Inequality_fam	0.333	0.048	0.239, 0.427	6.960	0.000

Supplementary Table 8

Regression estimates for the omitted Schwartz Values predicting the DFS

Outcome	Predictor	Standardised beta	SE	95% CI	Z	p-value
**Model 1 (N=489)**						
Inequality_nat	S_AC	0.053	0.053	-0.051, 0.157	1.003	0.316
	S_SDT	0.268	0.055	0.159, 0.376	4.847	0.000
	S_SDA	0.005	0.057	-0.106, 0.117	0.094	0.925
	S_TR	0.289	0.065	0.161, 0.416	4.446	0.000
	S_COR	0.008	0.056	-0.102, 0.119	0.151	0.880
**Model 2 (N=489)**						
Social_control_nat	S_SDA	0.040	0.053	-0.063, 0.144	0.762	0.446
	S_BEC	-0.105	0.066	-0.234, 0.023	-1.610	0.107
	S_BED	-0.089	0.058	-0.202, 0.024	-1.550	0.121
	S_UNC	0.182	0.058	0.067, 0.296	3.112	0.002
	S_AC	0.128	0.051	0.028, 0.228	2.510	0.012
**Model 3 (N=489)**						
Social_control_nat	S_SDT	0.092	0.058	-0.021, 0.204	1.593	0.111
	S_BEC	-0.123	0.066	-0.253, 0.007	-1.847	0.065
	S_BED	-0.091	0.057	-0.204, 0.021	-1.589	0.112
	S_UNC	0.164	0.059	0.047, 0.28	2.754	0.006
	S_AC	0.140	0.051	0.04, 0.24	2.750	0.006
**Model 4 (N=489)**						
Inequality_fam	S_AC	0.028	0.057	-0.084, 0.14	0.492	0.623
	S_SDT	0.309	0.059	0.194, 0.424	5.262	0.000
	S_SDA	0.060	0.061	-0.06, 0.179	0.978	0.328
	S_TR	0.064	0.072	-0.076, 0.205	0.901	0.367
	S_COR	0.000	0.061	-0.119, 0.119	0.000	1.000
**Model 5 (N=489)**						
Social_control_fam	S_SDA	0.134	0.051	0.034, 0.235	2.613	0.009
	S_BEC	-0.138	0.064	-0.264, -0.013	-2.162	0.031
	S_BED	-0.115	0.056	-0.225, -0.005	-2.050	0.040
	S_UNC	0.232	0.057	0.121, 0.343	4.086	0.000
	S_AC	0.122	0.050	0.024, 0.22	2.442	0.015
**Model 6 (N=489)**						
Social_control_fam	S_SDT	0.083	0.057	-0.028, 0.195	1.468	0.142
	S_BEC	-0.145	0.065	-0.273, -0.017	-2.214	0.027
	S_BED	-0.115	0.056	-0.225, -0.004	-2.032	0.042
	S_UNC	0.244	0.058	0.131, 0.357	4.228	0.000
	S_AC	0.108	0.050	0.009, 0.207	2.138	0.033

10 Preregistered analyses of Dictator Game Keepings using quantile regression

Our preregistration includes an analysis of Dictator Game Keepings as an outcome variable. However, since the distribution of Dictator Game keepings scores is clearly non-normal, this presents a challenge for modelling. We here use quantile regression because it is able to explore the effects of a predictor variable across multiple levels of the outcome variable. It works by testing whether relationships exist at different levels ('quantiles') of the outcome variable (Cade and Noon, 2003). We used the QuantReg package (Koenker, 2021) to perform these analyses. Because QuantReg does not support structural equation modelling, we used point estimates derived from factor analyses for the DFS dimensions, SDO, RWA, Nurturant Parent^{R}, and Strict Father. As in the main analyses, we controlled for participant sex, age, religion, education, and income.

We find that Dictator Game Keepings is predicted by DFS Nation Inequality (Supplementary Table 9) and DFS Family Inequality (Supplementary Table 10) at the 0.8 quantile. We also find that Dictator Game Keepings is predicted by SDO at the quantiles of 0.6 and 0.8 (Supplementary Table 11), and by Nurturant Parent^{R} at the 0.8 quantile (Supplementary Table 12).

Supplementary Table 9

Quantile regression estimates for the effects of DFS Nation Inequality and Social Control on Dictator Game Keepings

		DFS Nation Inequality	DFS Nation Social Control
**Quantile 0.0**			
	Estimate	0.000	0.000
	SE	0.223	0.208
	P-value	1.000	1.000
**Quantile 0.2**			
	Estimate	0.000	0.000
	SE	0.000	0.000
	P-value	0.418	0.801
**Quantile 0.4**			
	Estimate	0.000	0.000
	SE	0.000	0.000
	P-value	0.717	0.747
**Quantile 0.6**			
	Estimate	0.000	0.000
	SE	0.087	0.019
	P-value	1.000	1.000
**Quantile 0.8**			
	Estimate	0.845	-0.117
	SE	0.213	0.083
	P-value	0.000	0.156
**Quantile 1.0**			
	Estimate	0.000	0.000
	SE	0.208	0.252
	P-value	1.000	1.000

Supplementary Table 10

Quantile regression estimates for the effects of DFS Family Inequality and Social Control on Dictator Game Keepings

		DFS Family Inequality	DFS Family Social Control
**Quantile 0.0**			
	Estimate	-0.046	0.042
	SE	0.299	0.231
	P-value	0.879	0.856
**Quantile 0.2**			
	Estimate	0.000	0.000
	SE	0.000	0.000
	P-value	0.612	0.261
**Quantile 0.4**			
	Estimate	0.000	0.000
	SE	0.000	0.000
	P-value	0.841	0.992
**Quantile 0.6**			
	Estimate	0.000	0.000
	SE	0.043	0.027
	P-value	1.000	1.000
**Quantile 0.8**			
	Estimate	0.408	0.275
	SE	0.194	0.150
	P-value	0.036	0.068
**Quantile 1.0**			
	Estimate	0.000	0.000
	SE	0.158	0.185
	P-value	1.000	1.000

Supplementary Table 11

Quantile regression estimates for the effects of SDO and RWA on Dictator Game Keepings

	SDO	RWA
**Quantile 0.0**		
Estimate	-0.829	0.895
SE	0.366	0.363
P-value	0.024	0.014
**Quantile 0.2**		
Estimate	0.000	0.000
SE	0.000	0.000
P-value	0.334	0.884
**Quantile 0.4**		
Estimate	0.000	0.000
SE	0.000	0.000
P-value	0.244	0.914
**Quantile 0.6**		
Estimate	0.375	-0.027
SE	0.149	0.039
P-value	0.012	0.483
**Quantile 0.8**		
Estimate	0.786	-0.088
SE	0.095	0.096
P-value	0.000	0.364
**Quantile 1.0**		
Estimate	0.000	0.000
SE	0.326	0.211
P-value	1.000	1.000

Supplementary Table 12

Quantile regression estimates for the effects of Nurturant Parent (r) and Strict Father on Dictator Game Keepings

	Nurturant Parent (r)	Strict Father
**Quantile 0.0**		
Estimate	0.000	0.000
SE	0.439	0.223
P-value	1.000	1.000
**Quantile 0.2**		
Estimate	0.000	0.000
SE	0.000	0.000
P-value	0.788	0.614
**Quantile 0.4**		
Estimate	0.000	0.000
SE	0.000	0.000
P-value	0.462	0.613
**Quantile 0.6**		
Estimate	0.261	-0.005
SE	0.185	0.021
P-value	0.159	0.818
**Quantile 0.8**		
Estimate	0.743	0.153
SE	0.229	0.118
P-value	0.001	0.196
**Quantile 1.0**		
Estimate	0.000	0.000
SE	0.196	0.157
P-value	1.000	1.000

11 Pre-registered analyses of Rule Following using Bayesian multilevel modelling

We pre-registered analyses of Rule Following as an outcome variable, predicted by DFS Social Control. To take full advantage of the Rule Following data and respect the structure of the task, we employ a technique using stacked data to maximise the available information. The outcome variable represents each binary decision to either put the ball into the ‘rule following’ bucket or the payoff-maximising bucket. Each participant therefore has 30 outcome variables, one for each of their thirty binary decisions. This requires a multilevel approach, recognising that each individual’s thirty decisions are not independent and are ordered. We therefore created a multilevel Bayesian model using the brms package (Bürkner, 2017). To account for non-independence, the model contains random intercepts for participants alongside the fixed effects for predictors. To account for the ordered nature of participants’ decisions, the model contains a fixed effect for the round of the Rule Following task (from round 1 to 30). Since brms does not perform structural equation modelling, we used point estimates for DFS Social Control and Inequality produced by confirmatory factor analysis (as is true for the versions of the model that use SDO, RWA, Nurturant Parent^{R}, and Strict Father). All models also control for demographic covariates and religion. We standardised the ordinal and continuous predictor variables to have a mean of 0 and standard deviation of 1. We modelled the binary outcome using the Bernoulli distribution and used priors that assigned weight to values of 0 and 1. We used a prior predictive check to check the priors were reasonable. We obtained parameter estimates by running four Markov chains each for 20,000 iterations (half used for warm up). To confirm model convergence we ensured that chain reduction factors were at Rhat=1.00, which we accomplished by setting the sampler resolution to 0.9999 in the ‘adapt_delta’ argument of the ‘brm’ function. All parameters for the four models below have an effective sample size (measured by Bulk ESS and Tail ESS) of more than 2,500.

Supplementary Table 13 shows that DFS Nation Social Control positively predicts Rule Following, as does the Strict Father dimension. The greater part of the 95% confidence interval also lies above zero for DFS Family Social Control and RWA but in these cases crosses zero to include some negative values as well.

Supplementary Table 13				
Estimates for four Bayesian models predicting Rule Following with measures of social control, controlling for demographic covariates and religion				
Predictor	Estimate	Estimated Error	Lower 95% CI	Upper 95% CI
**Model 1 (N=489)**				
Round	0.077	0.133	-0.176	0.341
DFS Social Control Nation	0.655	0.309	0.049	1.262
DFS Inequality Nation	-0.338	0.305	-0.935	0.254
**Model 2 (N=489)**				
Round	0.093	0.135	-0.167	0.363
DFS Social Control Family	0.485	0.304	-0.109	1.082
DFS Inequality Family	0.166	0.298	-0.418	0.754
**Model 3 (N=489)**				
Round	0.055	0.135	-0.208	0.325
RWA	0.405	0.345	-0.278	1.077
SDO	-0.399	0.338	-1.053	0.266
**Model 4 (N=489)**				
Round	0.081	0.134	-0.178	0.349
SF	0.665	0.313	0.052	1.280
NP (r)	-0.174	0.308	-0.779	0.421

12 Pre-registered analyses of BEAST scores using Structural Equation Modelling

For this analysis, we use DFS data from waves one and two together. As shown in the main text, this composite DFS score demonstrates good internal validity. Supplementary Figures 4 and 5 show that we do not find that BEAST scores predict responses to the DFS, RWA, or the Strict Father dimension.

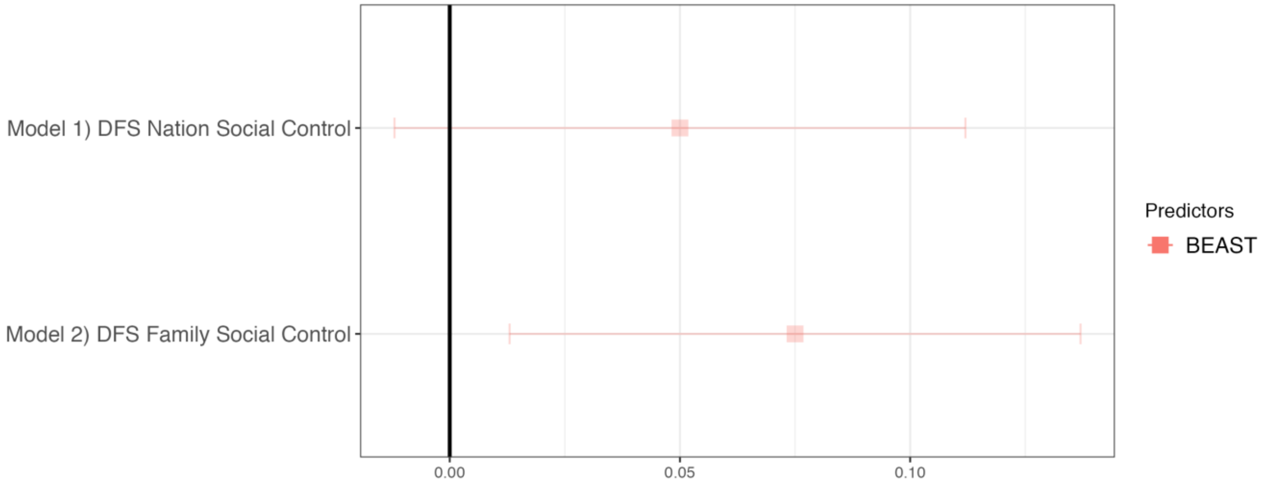

Supplementary Figure 4. The DFS does not relate to BEAST scores. Translucent points indicate p ≥ 0.05. Regression estimates are standardised betas with standard errors. Both models control for demographic covariates and religion.

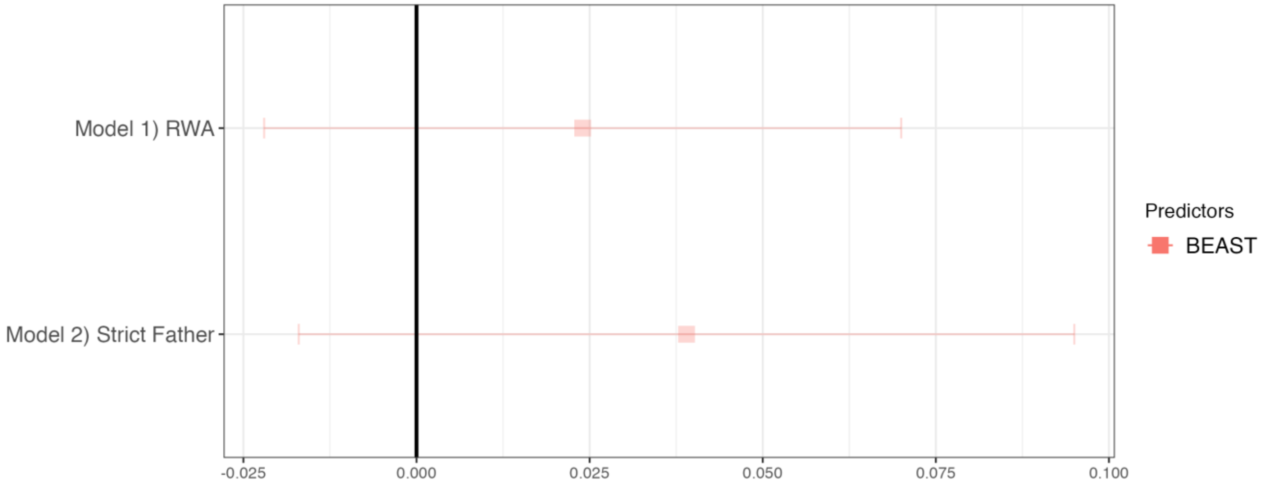

Supplementary Figure 5. Other measures of ideology also do not relate to BEAST scores. Translucent points indicate p ≥ 0.05. Regression estimates are standardised betas with standard errors. Both models control for demographic covariates and religion.

13 Pre-registered analyses of BEAST scores using Bayesian multilevel modelling

We pre-registered analyses of the BEAST as an outcome variable, predicted by DFS Social Control. To take full advantage of the BEAST data and respect the structure of the task, we employ a stacked data technique that maximises the available information. We created a multilevel Bayesian model with brms in which the outcome variable is the BEAST scores for each of the three rounds of the task. To account for the non-independence of each participant's three scores, the model contains random intercepts for participants as well as the fixed effects for predictors. To account for the ordered nature of participants' BEAST decisions, the model contains a fixed effect for round (one to three). We used point estimates from factor analysis of DFS Social Control and Inequality items combined across waves one and two, and point estimates from factor analysis based on wave 1 responses for SDO, RWA, Nurturant Parent^{R}, and Strict Father. We also controlled for demographic covariates and religion. We standardised our ordinal and continuous predictor variables to have a mean of 0 and standard deviation of 1. We scaled the outcome variable so that it varied between a minimum of 0 and a maximum of 1. We then modelled the outcome using the beta distribution and used priors which assigned roughly equal weight to values between 0 and 1. We used a prior predictive check to check the priors were reasonable. We obtained parameter estimates by running four Markov chains each for 20,000 iterations (half used for warm up). To confirm model convergence we ensured that chain reduction factors were at Rhat=1.00, which we accomplished by setting the sampler resolution to 0.9999 in the 'adapt_delta' argument of the 'brm' function. The parameters for the four models below all have an effective sample size (measured by Bulk ESS and Tail ESS) in excess 5,000.

Supplementary Table 14 shows that none of the scales of ideology had a consistent effect on BEAST scores. The 95% confidence intervals for the effects of DFS Social Control (Nation and Family), RWA, and Strict Father all cross zero to include both positive and negative values. Indeed, Supplementary Table 13 includes the covariates' estimates to show that none of the variables included in the models, besides the round of the task, reliably predicted BEAST scores. The negative effect of round in these models indicates that participants generally relied on social information more at the start of the task than the end. This lack of any effects, all with wide estimated errors, perhaps points to limited power to determine small effect sizes.

Supplementary Table 14

Estimates for four Bayesian models predicting BEAST scores with measures of social control

Predictor	Estimate	Estimated Error	Lower 95% CI	Upper 95% CI
**Model 1 (N=277)**				
Round	-0.228	0.078	-0.382	-0.078
DFS Nation Social Control	0.040	0.110	-0.176	0.256
DFS Nation Inequality	-0.106	0.108	-0.319	0.106
Men	0.104	0.197	-0.278	0.492
Age	-0.080	0.105	-0.287	0.125
Religion	0.161	0.204	-0.237	0.559
Education	0.020	0.105	-0.186	0.226
Income	-0.158	0.105	-0.363	0.047
**Model 2 (N=277)**				
Round	-0.284	0.075	-0.433	-0.140
DFS Family Social Control	0.109	0.104	-0.095	0.316
DFS Family Inequality	-0.173	0.101	-0.374	0.025
Men	-0.023	0.193	-0.401	0.355
Age	-0.074	0.104	-0.279	0.127
Religion	0.039	0.198	-0.351	0.430
Education	0.006	0.107	-0.203	0.216
Income	-0.167	0.104	-0.370	0.036
**Model 3 (N=277)**				
Round	-0.285	0.075	-0.434	-0.139
RWA	0.013	0.146	-0.270	0.302
SDO	-0.111	0.134	-0.375	0.153
Men	0.019	0.193	-0.358	0.400
Age	-0.074	0.106	-0.283	0.132
Religion	0.095	0.212	-0.317	0.512
Education	0.004	0.108	-0.207	0.219
Income	-0.165	0.103	-0.369	0.034
**Model 4 (N=277)**				
Round	-0.284	0.076	-0.434	-0.135
SF	0.081	0.116	-0.144	0.308
NP (r)	-0.074	0.109	-0.288	0.141
Men	0.001	0.197	-0.384	0.388
Age	-0.103	0.107	-0.314	0.108
Religion	0.025	0.205	-0.380	0.428
Education	0.025	0.108	-0.187	0.237
Income	-0.174	0.105	-0.378	0.031

14 Regression tables for figures 1 to 7 in the main text

Supplementary Table 15

Regression estimates for the four models in Figure 1

Outcome	Predictor	Standardised beta	SE	95% CI	Z	p-valu
**Model 1 (N=489)**						
Inequality_nat	Inequality_fam	0.840	0.033	0.775, 0.904	25.570	0.000
	Social_control_fam	0.119	0.045	0.031, 0.207	2.649	0.008
	Men	0.194	0.039	0.118, 0.27	5.017	0.000
	Age	0.082	0.040	0.003, 0.162	2.036	0.042
	Religion	0.107	0.039	0.031, 0.183	2.770	0.006
	Education	-0.005	0.042	-0.087, 0.077	-0.118	0.906
	Income	0.116	0.040	0.039, 0.194	2.939	0.003
**Model 2 (N=489)**						
Social_control_nat	Inequality_fam	0.112	0.048	0.018, 0.207	2.337	0.019
	Social_control_fam	0.699	0.035	0.631, 0.767	20.057	0.000
	Men	-0.016	0.041	-0.097, 0.066	-0.378	0.706
	Age	0.100	0.043	0.017, 0.184	2.352	0.019
	Religion	0.123	0.041	0.043, 0.203	3.025	0.002
	Education	-0.011	0.044	-0.098, 0.075	-0.256	0.798
	Income	0.075	0.042	-0.007, 0.157	1.789	0.074
**Model 3 (N=489)**						
Inequality_fam	Inequality_nat	0.870	0.033	0.805, 0.934	26.486	0.000
	Social_control_nat	-0.061	0.047	-0.153, 0.031	-1.296	0.195
	Men	-0.121	0.040	-0.199, -0.043	-3.039	0.002
	Age	-0.035	0.041	-0.116, 0.047	-0.835	0.404
	Religion	-0.054	0.040	-0.132, 0.023	-1.371	0.170
	Education	-0.034	0.043	-0.118, 0.049	-0.808	0.419
	Income	-0.042	0.041	-0.121, 0.038	-1.031	0.302
**Model 4 (N=489)**						
Social_control_fam	Inequality_nat	-0.040	0.049	-0.135, 0.056	-0.814	0.415
	Social_control_nat	0.697	0.037	0.626, 0.769	19.050	0.000
	Men	0.134	0.041	0.054, 0.215	3.271	0.001
	Age	-0.025	0.043	-0.109, 0.059	-0.580	0.562
	Religion	0.122	0.041	0.043, 0.202	3.005	0.003
	Education	-0.010	0.044	-0.097, 0.076	-0.232	0.816
	Income	0.035	0.042	-0.048, 0.117	0.826	0.409

Supplementary Table 16

Regression estimates for the four models in Figure 2

Outcome	Predictor	Standardised beta	SE	95% CI	Z	p-valu
**Model 1 (N=489)**						
Pol_orient	Inequality_nat	0.443	0.040	0.365, 0.521	11.143	0.000
	Social_control_nat	0.250	0.041	0.169, 0.332	6.034	0.000
	Men	0.124	0.037	0.052, 0.196	3.364	0.001
	Age	0.134	0.038	0.06, 0.209	3.531	0.000
	Religion	0.250	0.035	0.18, 0.319	7.060	0.000
	Education	-0.100	0.039	-0.177, -0.023	-2.532	0.011
	Income	0.083	0.037	0.009, 0.156	2.208	0.027
**Model 2 (N=489)**						
Pol_orient	Inequality_fam	0.326	0.043	0.242, 0.411	7.578	0.000
	Social_control_fam	0.291	0.041	0.21, 0.372	7.039	0.000
	Men	0.169	0.038	0.094, 0.243	4.430	0.000
	Age	0.176	0.039	0.099, 0.253	4.482	0.000
	Religion	0.284	0.036	0.213, 0.355	7.822	0.000
	Education	-0.098	0.041	-0.179, -0.018	-2.389	0.017
	Income	0.127	0.039	0.05, 0.203	3.257	0.001
**Model 3 (N=489)**						
Pol_orient	SDO	0.319	0.043	0.235, 0.403	7.438	0.000
	RWA	0.440	0.041	0.359, 0.521	10.630	0.000
	Men	0.074	0.033	0.009, 0.138	2.246	0.025
	Age	0.075	0.034	0.009, 0.142	2.215	0.027
	Religion	0.089	0.033	0.025, 0.152	2.727	0.006
	Education	-0.035	0.035	-0.103, 0.034	-0.984	0.325
	Income	0.112	0.033	0.047, 0.177	3.377	0.001
**Model 4 (N=489)**						
Pol_orient	Nurturant_parent	0.221	0.043	0.137, 0.304	5.162	0.000
	Strict_father	0.367	0.040	0.289, 0.445	9.190	0.000
	Men	0.112	0.038	0.037, 0.187	2.935	0.003
	Age	0.120	0.039	0.043, 0.197	3.049	0.002
	Religion	0.193	0.037	0.12, 0.266	5.192	0.000
	Education	-0.090	0.041	-0.17, -0.01	-2.207	0.027
	Income	0.138	0.039	0.062, 0.213	3.576	0.000

Supplementary Table 17

Regression estimates for the eight models in Figure 3

Outcome	Predictor	Standardised beta	SE	95% CI	Z	p-value
**Model 1 (N=489)**						
Inequality_nat	SDO	0.839	0.044	0.754, 0.925	19.178	0.000
	RWA	-0.124	0.054	-0.229, -0.019	-2.315	0.021
	Men	0.045	0.040	-0.034, 0.125	1.124	0.261
	Age	0.016	0.042	-0.065, 0.098	0.395	0.693
	Religion	0.015	0.040	-0.064, 0.093	0.363	0.716
	Education	0.029	0.043	-0.055, 0.114	0.682	0.495
	Income	0.091	0.041	0.011, 0.171	2.236	0.025
**Model 2 (N=489)**						
Social_control_nat	RWA	0.752	0.047	0.661, 0.844	16.053	0.000
	SDO	-0.212	0.056	-0.321, -0.103	-3.813	0.000
	Men	0.019	0.042	-0.063, 0.101	0.458	0.647
	Age	0.011	0.043	-0.074, 0.096	0.256	0.798
	Religion	-0.010	0.042	-0.091, 0.072	-0.240	0.811
	Education	0.038	0.045	-0.05, 0.125	0.844	0.399
	Income	0.116	0.042	0.033, 0.198	2.741	0.006
**Model 3 (N=489)**						
SDO	Inequality_nat	0.734	0.031	0.673, 0.795	23.544	0.000
	Social_control_nat	0.075	0.041	-0.006, 0.156	1.812	0.070
	Men	0.128	0.035	0.059, 0.197	3.647	0.000
	Age	0.069	0.036	-0.002, 0.141	1.894	0.058
	Religion	0.128	0.035	0.06, 0.196	3.678	0.000
	Education	-0.088	0.038	-0.161, -0.014	-2.333	0.020
	Income	0.003	0.036	-0.067, 0.073	0.090	0.928
**Model 4 (N=489)**						
RWA	Social_control_nat	0.496	0.037	0.425, 0.568	13.553	0.000
	Inequality_nat	0.251	0.041	0.17, 0.331	6.101	0.000
	Men	0.083	0.036	0.013, 0.153	2.335	0.020
	Age	0.133	0.037	0.061, 0.205	3.635	0.000
	Religion	0.352	0.033	0.288, 0.417	10.754	0.000
	Education	-0.110	0.038	-0.184, -0.036	-2.912	0.004
	Income	-0.028	0.036	-0.099, 0.043	-0.774	0.439
**Model 5 (N=489)**						
Inequality_fam	SDO	0.662	0.056	0.552, 0.771	11.807	0.000
	RWA	-0.215	0.062	-0.337, -0.093	-3.444	0.001
	Men	-0.100	0.047	-0.192, -0.007	-2.110	0.035
	Age	-0.009	0.049	-0.105, 0.088	-0.177	0.860
	Religion	-0.016	0.047	-0.108, 0.076	-0.341	0.733
	Education	-0.022	0.051	-0.121, 0.077	-0.434	0.664
	Income	0.016	0.048	-0.079, 0.11	0.324	0.746
**Model 6 (N=489)**						
Social_control_fam	RWA	0.553	0.053	0.448, 0.658	10.343	0.000
	SDO	-0.020	0.059	-0.135, 0.096	-0.334	0.739
	Men	0.097	0.044	0.011, 0.184	2.214	0.027
	Age	-0.058	0.046	-0.148, 0.031	-1.279	0.201
	Religion	0.027	0.044	-0.059, 0.113	0.620	0.536
	Education	0.037	0.047	-0.055, 0.13	0.795	0.427
	Income	0.077	0.045	-0.01, 0.165	1.732	0.083
**Model 7 (N=489)**						
SDO	Inequality_fam	0.519	0.041	0.44, 0.599	12.757	0.000
	Social_control_fam	0.295	0.043	0.212, 0.379	6.916	0.000
	Men	0.206	0.038	0.131, 0.281	5.393	0.000
	Age	0.109	0.040	0.03, 0.188	2.704	0.007
	Religion	0.157	0.038	0.082, 0.232	4.090	0.000
	Education	-0.082	0.042	-0.164, -0.001	-1.975	0.048
	Income	0.053	0.040	-0.024, 0.131	1.347	0.178
**Model 8 (N=489)**						
RWA	Social_control_fam	0.468	0.038	0.394, 0.542	12.451	0.000
	Inequality_fam	0.179	0.043	0.094, 0.263	4.122	0.000
	Men	0.092	0.037	0.019, 0.166	2.456	0.014
	Age	0.183	0.038	0.108, 0.258	4.791	0.000
	Religion	0.385	0.034	0.319, 0.452	11.440	0.000
	Education	-0.112	0.040	-0.19, -0.034	-2.815	0.005
	Income	0.013	0.038	-0.062, 0.088	0.342	0.732

Supplementary Table 18

Regression estimates for the eight models in Figure 4

Outcome	Predictor	Standardised beta	SE	95% CI	Z	p-value
**Model 1 (N=489)**						
Inequality_fam	Nurturant_parent	0.620	0.045	0.533, 0.708	13.910	0.000
	Strict_father	-0.052	0.051	-0.153, 0.049	-1.014	0.311
	Men	-0.119	0.046	-0.21, -0.028	-2.572	0.010
	Age	0.008	0.048	-0.087, 0.102	0.162	0.871
	Religion	-0.026	0.046	-0.116, 0.065	-0.553	0.580
	Education	-0.034	0.050	-0.131, 0.064	-0.680	0.497
	Income	0.059	0.047	-0.034, 0.151	1.244	0.214
**Model 2 (N=489)**						
Social_control_fam	Strict_father	0.553	0.042	0.471, 0.635	13.249	0.000
	Nurturant_parent	0.036	0.050	-0.062, 0.133	0.715	0.474
	Men	0.093	0.044	0.007, 0.179	2.117	0.034
	Age	-0.064	0.045	-0.153, 0.025	-1.415	0.157
	Religion	0.058	0.044	-0.027, 0.143	1.330	0.184
	Education	0.004	0.047	-0.088, 0.096	0.089	0.929
	Income	0.063	0.044	-0.024, 0.15	1.416	0.157
**Model 3 (N=489)**						
Nurturant_parent	Inequality_fam	0.592	0.041	0.511, 0.673	14.344	0.000
	Social_control_fam	0.213	0.046	0.123, 0.303	4.647	0.000
	Men	0.242	0.040	0.163, 0.321	6.021	0.000
	Age	0.024	0.043	-0.06, 0.108	0.555	0.579
	Religion	0.083	0.041	0.003, 0.164	2.024	0.043
	Education	-0.038	0.044	-0.125, 0.049	-0.858	0.391
	Income	-0.040	0.042	-0.122, 0.043	-0.949	0.343
**Model 4 (N=489)**						
Strict_father	Social_control_fam	0.508	0.038	0.433, 0.583	13.248	0.000
	Inequality_fam	0.132	0.046	0.043, 0.221	2.903	0.004
	Men	0.081	0.039	0.004, 0.158	2.063	0.039
	Age	0.194	0.040	0.115, 0.272	4.851	0.000
	Religion	0.314	0.037	0.242, 0.386	8.560	0.000
	Education	-0.042	0.042	-0.125, 0.04	-1.005	0.315
	Income	0.044	0.040	-0.034, 0.122	1.106	0.269
**Model 5 (N=489)**						
Inequality_nat	Nurturant_parent	0.597	0.043	0.512, 0.681	13.882	0.000
	Strict_father	0.134	0.048	0.039, 0.229	2.756	0.006
	Men	0.061	0.044	-0.026, 0.147	1.370	0.171
	Age	0.052	0.046	-0.037, 0.142	1.144	0.253
	Religion	0.023	0.044	-0.063, 0.109	0.518	0.605
	Education	-0.003	0.047	-0.095, 0.09	-0.056	0.955
	Income	0.150	0.044	0.063, 0.236	3.372	0.001
**Model 6 (N=489)**						
Social_control_nat	Strict_father	0.601	0.040	0.522, 0.68	14.934	0.000
	Nurturant_parent	-0.019	0.050	-0.116, 0.078	-0.393	0.694
	Men	0.004	0.044	-0.082, 0.09	0.095	0.924
	Age	0.012	0.045	-0.077, 0.1	0.263	0.793
	Religion	0.054	0.043	-0.031, 0.138	1.237	0.216
	Education	-0.001	0.047	-0.093, 0.09	-0.030	0.976
	Income	0.092	0.044	0.006, 0.179	2.089	0.037
**Model 7 (N=489)**						
Nurturant_parent	Inequality_nat	0.621	0.040	0.542, 0.7	15.378	0.000
	Social_control_nat	0.037	0.048	-0.057, 0.13	0.771	0.441
	Men	0.174	0.041	0.094, 0.254	4.251	0.000
	Age	-0.012	0.043	-0.096, 0.072	-0.277	0.782
	Religion	0.059	0.041	-0.022, 0.139	1.432	0.152
	Education	-0.050	0.044	-0.137, 0.037	-1.135	0.256
	Income	-0.080	0.042	-0.162, 0.003	-1.896	0.058
**Model 8 (N=489)**						
Strict_father	Social_control_nat	0.504	0.039	0.427, 0.58	12.850	0.000
	Inequality_nat	0.193	0.045	0.105, 0.28	4.306	0.000
	Men	0.088	0.039	0.012, 0.163	2.269	0.023
	Age	0.148	0.040	0.07, 0.225	3.741	0.000
	Religion	0.294	0.036	0.223, 0.365	8.082	0.000
	Education	-0.040	0.041	-0.121, 0.041	-0.966	0.334
	Income	0.012	0.039	-0.065, 0.089	0.305	0.760

Supplementary Table 19

Regression estimates for the ten models in Figure 5

Outcome	Predictor	Standardised beta	SE	95% CI	Z	p-value
**Model 1 (N=489)**						
S_BEC	Inequality_nat	0.368	0.047	0.276, 0.461	7.797	0.000
	Social_control_nat	-0.126	0.049	-0.222, -0.031	-2.595	0.009
	Men	0.051	0.043	-0.034, 0.135	1.177	0.239
	Age	-0.075	0.044	-0.162, 0.012	-1.686	0.092
	Religion	-0.031	0.043	-0.115, 0.052	-0.736	0.462
	Education	-0.041	0.046	-0.131, 0.049	-0.888	0.375
	Income	-0.018	0.044	-0.104, 0.068	-0.407	0.684
**Model 2 (N=489)**						
S_BED	Inequality_nat	0.183	0.051	0.082, 0.283	3.554	0.000
	Social_control_nat	-0.131	0.050	-0.23, -0.032	-2.596	0.009
	Men	-0.047	0.045	-0.135, 0.041	-1.042	0.297
	Age	0.009	0.047	-0.082, 0.1	0.197	0.843
	Religion	-0.044	0.045	-0.131, 0.043	-0.987	0.324
	Education	-0.020	0.048	-0.114, 0.074	-0.419	0.675
	Income	-0.030	0.046	-0.119, 0.06	-0.653	0.514
**Model 3 (N=489)**						
S_UNC	Inequality_nat	0.555	0.040	0.477, 0.632	14.029	0.000
	Social_control_nat	0.030	0.045	-0.059, 0.118	0.658	0.510
	Men	0.085	0.039	0.008, 0.163	2.171	0.030
	Age	0.004	0.041	-0.076, 0.084	0.104	0.918
	Religion	0.013	0.039	-0.064, 0.089	0.319	0.750
	Education	-0.082	0.042	-0.164, 0	-1.950	0.051
	Income	-0.012	0.040	-0.091, 0.066	-0.303	0.762
**Model 4 (N=489)**						
S_TR	Social_control_nat	0.304	0.040	0.226, 0.381	7.679	0.000
	Inequality_nat	0.174	0.042	0.093, 0.256	4.187	0.000
	Men	0.041	0.036	-0.03, 0.112	1.141	0.254
	Age	0.130	0.037	0.057, 0.202	3.511	0.000
	Religion	0.477	0.030	0.418, 0.536	15.815	0.000
	Education	0.054	0.038	-0.021, 0.129	1.403	0.161
	Income	-0.017	0.037	-0.089, 0.055	-0.456	0.648
**Model 5 (N=489)**						
S_COR	Social_control_nat	0.521	0.038	0.446, 0.596	13.578	0.000
	Inequality_nat	0.054	0.045	-0.035, 0.143	1.191	0.234
	Men	-0.072	0.039	-0.148, 0.004	-1.856	0.063
	Age	0.118	0.040	0.04, 0.195	2.958	0.003
	Religion	0.170	0.038	0.096, 0.244	4.519	0.000
	Education	0.002	0.041	-0.079, 0.083	0.045	0.964
	Income	0.009	0.039	-0.068, 0.086	0.239	0.811
**Model 6 (N=489)**						
S_BEC	Inequality_fam	0.407	0.045	0.318, 0.496	8.997	0.000
	Social_control_fam	-0.032	0.047	-0.124, 0.06	-0.687	0.492
	Men	0.103	0.042	0.02, 0.185	2.433	0.015
	Age	-0.063	0.044	-0.149, 0.023	-1.444	0.149
	Religion	-0.016	0.042	-0.098, 0.067	-0.375	0.707
	Education	-0.031	0.045	-0.12, 0.058	-0.683	0.495
	Income	0.001	0.043	-0.083, 0.086	0.033	0.974
**Model 7 (N=489)**						
S_BED	Inequality_fam	0.156	0.051	0.056, 0.257	3.042	0.002
	Social_control_fam	-0.101	0.049	-0.197, -0.005	-2.060	0.039
	Men	-0.014	0.045	-0.102, 0.075	-0.306	0.760
	Age	0.012	0.047	-0.079, 0.104	0.265	0.791
	Religion	-0.032	0.045	-0.119, 0.056	-0.711	0.477
	Education	-0.018	0.048	-0.112, 0.077	-0.369	0.712
	Income	-0.018	0.046	-0.108, 0.071	-0.403	0.687
**Model 8 (N=489)**						
S_UNC	Inequality_fam	0.487	0.042	0.405, 0.569	11.637	0.000
	Social_control_fam	0.209	0.044	0.123, 0.296	4.739	0.000
	Men	0.146	0.040	0.068, 0.224	3.668	0.000
	Age	0.035	0.042	-0.047, 0.117	0.839	0.401
	Religion	0.032	0.040	-0.046, 0.11	0.799	0.424
	Education	-0.074	0.043	-0.158, 0.01	-1.716	0.086
	Income	0.023	0.041	-0.057, 0.103	0.556	0.578
**Model 9 (N=489)**						
S_TR	Social_control_fam	0.356	0.038	0.282, 0.429	9.460	0.000
	Inequality_fam	0.060	0.042	-0.022, 0.141	1.434	0.151
	Men	0.042	0.036	-0.029, 0.112	1.150	0.250
	Age	0.160	0.037	0.088, 0.232	4.362	0.000
	Religion	0.484	0.030	0.425, 0.543	16.177	0.000
	Education	0.050	0.038	-0.025, 0.125	1.299	0.194
	Income	0.007	0.037	-0.065, 0.079	0.193	0.847
**Model 10 (N=489)**						
S_COR	Social_control_fam	0.353	0.043	0.269, 0.436	8.283	0.000
	Inequality_fam	0.061	0.048	-0.032, 0.155	1.288	0.198
	Men	-0.074	0.041	-0.155, 0.007	-1.786	0.074
	Age	0.159	0.042	0.077, 0.242	3.782	0.000
	Religion	0.213	0.040	0.135, 0.291	5.368	0.000
	Education	-0.002	0.044	-0.089, 0.084	-0.051	0.959
	Income	0.043	0.042	-0.04, 0.125	1.016	0.310

Supplementary Table 20

Regression estimates for the four models in Figure 6

Outcome	Predictor	Standardised beta	SE	95% CI	Z	p-valu
**Model 1 (N=489)**						
Inequality_nat	DG_keepings	0.203	0.049	0.108, 0.299	4.158	0.000
	Rule_following	-0.008	0.050	-0.107, 0.09	-0.168	0.867
	Social_control_nat	0.117	0.054	0.013, 0.222	2.195	0.028
	Men	0.182	0.049	0.085, 0.278	3.694	0.000
	Age	0.088	0.051	-0.011, 0.187	1.734	0.083
	Religion	0.137	0.049	0.042, 0.233	2.824	0.005
	Education	-0.029	0.053	-0.132, 0.074	-0.552	0.581
	Income	0.132	0.050	0.034, 0.229	2.648	0.008
**Model 2 (N=489)**						
Social_control_nat	Rule_following	0.130	0.049	0.034, 0.226	2.655	0.008
	DG_keepings	0.052	0.049	-0.045, 0.148	1.048	0.295
	Inequality_nat	0.120	0.054	0.014, 0.226	2.221	0.026
	Men	0.073	0.049	-0.023, 0.169	1.488	0.137
	Age	0.116	0.050	0.019, 0.214	2.343	0.019
	Religion	0.240	0.047	0.149, 0.331	5.166	0.000
	Education	-0.029	0.052	-0.13, 0.073	-0.557	0.578
	Income	0.130	0.049	0.034, 0.226	2.659	0.008
**Model 3 (N=489)**						
Inequality_fam	DG_keepings	0.155	0.052	0.053, 0.258	2.965	0.003
	Rule_following	0.087	0.053	-0.018, 0.191	1.630	0.103
	Social_control_fam	-0.124	0.056	-0.235, -0.014	-2.202	0.028
	Men	0.031	0.053	-0.073, 0.135	0.578	0.563
	Age	0.032	0.054	-0.074, 0.138	0.599	0.549
	Religion	0.060	0.052	-0.042, 0.162	1.151	0.250
	Education	-0.064	0.056	-0.173, 0.046	-1.139	0.255
	Income	0.062	0.053	-0.042, 0.166	1.166	0.244
**Model 4 (N=489)**						
Social_control_fam	Rule_following	0.104	0.048	0.009, 0.198	2.152	0.031
	DG_keepings	0.160	0.047	0.067, 0.253	3.380	0.001
	Inequality_fam	-0.113	0.054	-0.218, -0.007	-2.087	0.037
	Men	0.164	0.047	0.071, 0.257	3.453	0.001
	Age	0.045	0.049	-0.051, 0.141	0.920	0.357
	Religion	0.255	0.045	0.166, 0.344	5.633	0.000
	Education	-0.026	0.051	-0.125, 0.074	-0.506	0.613
	Income	0.107	0.048	0.013, 0.201	2.233	0.026

Supplementary Table 21

Regression estimates for the four models in Figure 7

Outcome	Predictor	Standardised beta	SE	95% CI	Z	p-valu
**Model 1 (N=489)**						
SDO	DG_keepings	0.220	0.037	0.148, 0.292	5.978	0.000
	Rule_following	-0.052	0.038	-0.127, 0.022	-1.382	0.167
	RWA	0.578	0.031	0.517, 0.639	18.547	0.000
	Men	0.133	0.037	0.06, 0.207	3.571	0.000
	Age	0.015	0.038	-0.06, 0.09	0.389	0.697
	Religion	-0.010	0.037	-0.083, 0.062	-0.274	0.784
	Education	-0.027	0.040	-0.105, 0.051	-0.678	0.498
	Income	0.048	0.038	-0.026, 0.122	1.279	0.201
**Model 2 (N=489)**						
RWA	Rule_following	0.079	0.037	0.007, 0.151	2.157	0.031
	DG_keepings	0.044	0.037	-0.028, 0.115	1.193	0.233
	SDO	0.537	0.032	0.474, 0.599	16.900	0.000
	Men	0.042	0.037	-0.029, 0.114	1.154	0.248
	Age	0.141	0.037	0.069, 0.214	3.849	0.000
	Religion	0.370	0.033	0.306, 0.435	11.260	0.000
	Education	-0.076	0.038	-0.151, -0.001	-1.985	0.047
	Income	0.014	0.037	-0.058, 0.086	0.381	0.703
**Model 3 (N=489)**						
Nurturant_parent	DG_keepings	0.187	0.046	0.097, 0.277	4.068	0.000
	Rule_following	0.015	0.047	-0.078, 0.107	0.312	0.755
	Strict_father	0.240	0.046	0.15, 0.331	5.207	0.000
	Men	0.223	0.045	0.134, 0.312	4.908	0.000
	Age	-0.011	0.048	-0.104, 0.083	-0.222	0.824
	Religion	0.055	0.046	-0.035, 0.145	1.205	0.228
	Education	-0.046	0.049	-0.143, 0.05	-0.941	0.347
	Income	-0.032	0.047	-0.124, 0.06	-0.678	0.498
**Model 4 (N=489)**						
Strict_father	Rule_following	0.135	0.042	0.052, 0.217	3.201	0.001
	DG_keepings	0.122	0.042	0.04, 0.204	2.905	0.004
	Nurturant_parent	0.208	0.043	0.123, 0.293	4.799	0.000
	Men	0.105	0.042	0.023, 0.188	2.507	0.012
	Age	0.202	0.042	0.12, 0.284	4.821	0.000
	Religion	0.378	0.037	0.304, 0.451	10.072	0.000
	Education	-0.041	0.044	-0.128, 0.046	-0.915	0.360
	Income	0.092	0.042	0.009, 0.175	2.185	0.029

15 References

Bürkner, P.-C. (2017) ‘brms: An R Package for Bayesian Multilevel Models Using Stan’, *Journal of Statistical Software*, 80(1), pp. 1–28. Available at: https://doi.org/10.18637/jss.v080.i01.

Cade, B.S. and Noon, B.R. (2003) ‘A Gentle Introduction to Quantile Regression for Ecologists’, *Frontiers in Ecology and the Environment*, 1(8), p. 412. Available at: https://doi.org/10.2307/3868138.

Claessens, S. *et al.* (2021) ‘Cooperative and conformist behavioural preferences predict the dual dimensions of political ideology’, *PsyArXiv* [Preprint]. Available at: https://doi.org/10.31234/osf.io/t7rqb.

Koenker, R. (2021) *Package ‘quantreg’*. [if not showing URL add manually]

Molleman, L., Kurvers, R.H.J.M. and van den Bos, W. (2019) ‘Unleashing the BEAST: a brief measure of human social information use’, *Evolution and Human Behavior*, 40(5), pp. 492–499. Available at: https://doi.org/10.1016/j.evolhumbehav.2019.06.005.
